# Co-ordinate regulation of cytokinin gene family members during flag leaf and reproductive development in wheat

**DOI:** 10.1186/1471-2229-12-78

**Published:** 2012-06-06

**Authors:** Jiancheng Song, Lijun Jiang, Paula Elizabeth Jameson

**Affiliations:** 1 School of Biological Sciences, University of Canterbury, Private Bag 4800, Christchurch, New Zealand; 2 School of Life Sciences, Yantai University, 32 Qingquan Road, Yantai, 264005, China

## Abstract

**Background:**

As the global population continues to expand, increasing yield in bread wheat is of critical importance as 20% of the world’s food supply is sourced from this cereal. Several recent studies of the molecular basis of grain yield indicate that the cytokinins are a key factor in determining grain yield. In this study, cytokinin gene family members in bread wheat were isolated from four multigene families which regulate cytokinin synthesis and metabolism, the isopentenyl transferases (*IPT*), cytokinin oxidases (*CKX*), zeatin *O*-glucosyltransferases (*ZOG*), and β-glucosidases (*GLU*). As bread wheat is hexaploid, each gene family is also likely to be represented on the A, B and D genomes. By using a novel strategy of qRT-PCR with locus-specific primers shared among the three homoeologues of each family member, detailed expression profiles are provided of family members of these multigene families expressed during leaf, spike and seed development.

**Results:**

The expression patterns of individual members of the *IPT*, *CKX*, *ZOG*, and *GLU* multigene families in wheat are shown to be tissue- and developmentally-specific. For instance, *TaIPT2* and *TaCKX1* were the most highly expressed family members during early seed development, with relative expression levels of up to 90- and 900-fold higher, respectively, than those in the lowest expressed samples. The expression of two cis-*ZOG* genes was sharply increased in older leaves, while an extremely high mRNA level of *TaGLU1-1* was detected in young leaves.

**Conclusions:**

Key genes with tissue- and developmentally-specific expression have been identified which would be prime targets for genetic manipulation towards yield improvement in bread wheat breeding programmes, utilising TILLING and MAS strategies.

## Background

Bread wheat (*Triticum aestivum* L.) is one of the world’s most important crop plants with a total production of over 600 million tons in 2007 [1]. This accounts for over 20% of the world’s food supply. The increase in the global population is expected to increase the demand for wheat to over 800 million tons in 2030 and to more than 900 million tons by 2050 [2]. Meeting this demand is predicted to depend largely on increasing yield through genetic improvement. However, in recent decades, internationally coordinated public wheat breeding efforts have focused on increasing resistance to disease and abiotic stress, while raising the genetic yield potential *per se* has received scant attention. Moreover, the challenges of increasing the genetic yield potential are considerable [3].

Grain size and grain number are significant components of yield in cereals, and both are major contributors to seed sink strength [4]. Yet, until recently, our understanding of yield at the molecular level was limited and little progress has been made towards the identification of the genetic components that define yield in a quantitative manner [5]. Some of the most significant recent studies towards the understanding of the molecular basis of grain size and number include identification of a number of genes and quantitative trait loci (QTLs) contributing to seed number and size in *Arabidopsis*[6,7], rice [8,9] and maize [10], and the recognition that these genes and/or QTLs are cytokinin synthesis and metabolism genes.

Cytokinins (CKs) are hormones that regulate many developmental and physiological processes in plants [11], including reproductive development and grain yield [7,8,12,13]. In general, cytokinins may affect seed yield by enhancing sink strength and, consequently, increasing seed size, by up-regulating cell cycle related genes, through the action of sugar signalling and by enhancing phloem unloading and sugar import into the endosperm through enzymatic activity of cell wall invertase [12]. Although the beneficial effect of exogenously applied cytokinins on grain yield has been documented for several decades, including for wheat, maize, rice and barley [14-17], the application of cytokinins to field crops is limited due to the substantial cost of cytokinins. Given that changes in the levels of endogenous cytokinins will alter the regulation of many physiological processes resulting in the disruption of normal growth patterns [18], and rapid and dramatic changes in cytokinin concentration have been observed during panicle and early grain development in a number of crop species including wheat [19-21] and rice [8,22], yield improvement through genetic manipulation of cytokinin synthesis and metabolism genes to artificially manipulate the endogenous cytokinin levels could be of considerable importance.

The last decade has seen the key steps in cytokinin biosynthesis, metabolism, and signal transduction elucidated at the molecular level [23]. The bioactive cytokinin concentration is regulated by several multigene families, including isopentenyl transferases (*IPT*) for cytokinin biosynthesis, cytokinin oxidases (*CKX*) for degradation, zeatin *O*-glucosyltransferases (*ZOG*) for reversible inactivation, and β-glucosidases (*GLU*) for reactivation. In *Arabidopsis*, nine *IPT* genes, seven *CKX* genes, three *ZOG* genes and *GLU* genes have been identified and functionally verified [24-28]. Their orthologues have also been annotated and/or functionally verified in a number of important crop species. In rice, for instance, eight *IPTs* and 11 *CKXs* have been isolated [29-31]. Similarly, 11 *IPTs*, 13 *CKXs*, and several *ZOG*s and *GLUs* have been isolated from maize [32-34].

Because of the multigene family nature of these cytokinin metabolism genes, each member can be functionally differentiated based on their spatial and temporal expression patterns [31]. Indeed, the temporal and spatial expression, and functional analysis, of specific family members has been confirmed in a number of species. For example, in *Zea mays,* the temporal and spatial expression, and functional analysis, of specific gene family members*,* confirmed that maize seed biosynthesises its own endogenous cytokinin, and that *ZmIPT2* and *ZmCKX1* are the gene family members controlling cytokinin homeostasis in the developing maize grain [32,35]. In rice, Ashikari *et al.*[8] showed that a QTL associated with increased grain productivity was in fact a mutated cytokinin oxidase gene, *OsCKX2*. Most recently, RNAi was used to silence *HvCKX1* in barley with a resultant increase in both grain number and grain weight [13].

Due to the pleiotropic nature of cytokinins and the variation in spatial expression of gene family members, manipulation of a particular gene may cause side effects, affecting non target organs or developmental stages. Therefore, detailed expression profiles of all available members of the major gene families involved in cytokinin synthesis and metabolism (i.e. the *IPTsCKXsZOGs* and *GLUs*), should be investigated simultaneously in order to determine the key genes with critical roles in the target organ or developmental stage. While there are examples in the literature showing that there is variation in the spatial and temporal expression patterns of individual cytokinin gene family members [32,36-41], simultaneous monitoring of the four major cytokinin metabolism gene families has only recently been reported in maize [33], but without detailed information during leaf, floral and seed ontology.

Apart from our preliminary report [41], detailed information regarding the expression profiles and functional territories of these multigene families is lacking for bread wheat, possibly due to the added complexity of its hexaploid nature, such that each gene is represented in triplicate homoeologues on the three genomes [42]. Further, each triplicate homoeologue within the same locus can be differentially regulated by genetic and epigenetic mechanisms in a tissue and developmentally specific manner [43-45]. In general, uniformly expressed homoeologues only account for 20-40% of the loci in bread wheat, with 20-30% of loci presenting at least one silenced homoeologue and, for the rest of the loci, the three homoeologues are differentially expressed across organs and developmental stages [43-46].

Given the above, we hypothesised that cytokinin homeostasis within an organ is co-ordinately regulated by particular homoeologues of different gene family members to allow the precise control of organ development, and that optimum seed yield could be obtained by manipulating the endogenous cytokinin concentration at specific stages of reproductive growth and seed development through genetic manipulation utilising, for example, over-expression, gene silencing, TILLING and MAS techniques.

However, it would be very complicated and essentially impractical to determine the expression profile of each homoeologue of each gene family member simultaneously, particularly when multiple tissue types and time points are involved. Due to this complexity only three *TaCKX* genes [47-49] and one set of *GLU* homoeologous genes [50] have been isolated and investigated so far, with little information regarding their expression profiles. No study with regard to *IPT* and *ZOG* genes has yet been reported for wheat.

In this study, we report the isolation and simultaneous expression analysis of *TaIPT*, *TaCKX*, *TaZOG* and *TaGLU* gene family members during flag leaf, spike and seed development by using a novel strategy in which locus-specific PCR primers shared by the three homoeologues were used, and reveal the co-ordinated expression of several members of different multigene families.

## Methods

### Plant material

Winter bread wheat, variety Equinox, was grown under prevailing climatic conditions in a nursery plot at the New Zealand Institute for Plant & Food Research, Lincoln, Canterbury, New Zealand. After microscopic determination, representative developmental stages of spikes, carpels, seeds and the basal one-third of flag leaves were harvested, from spikelet initiation to 28 days after anthesis (daa) and immediately frozen in liquid nitrogen and stored at −80 °C until used. The chlorophyll content of flag leaf samples was determined as described in O’Keefe *et al.*[40]. The total chlorophyll content was calculated using the equations of Porra [51]:

(1) $$\text{Chl}\;a+b=17.67{\text{Abs}}_{647}+7.{12\text{Abs}}_{664}\text{.}$$

### RNA extraction and cDNA synthesis

Total RNA was extracted using TRIzol Reagent (Invitrogen, Carlsbad, CA, USA) following the manufacturer’s protocol. Two independent tissue samples of each developmental stage were used as biological replicates. The concentration and purity of the total RNA extracted was determined using a Nanodrop spectrophotometer (Nanodrop Technologies Inc, Wilmington, DE, USA). The integrity of the total RNA was determined by electrophoresis on 1% (w/v) agarose gels. For cDNA synthesis, l μg total RNA, 50 U Expand Reverse Transcriptase (Roche, Mannheim, Germany), 50 pmol oligo (dT) primers and 100 pmol random hexamer (pdN6) primers were used in a 20 μl reverse transcription reaction. The final reaction mix was incubated at 42 °C for 2 h.

### Gene isolation and sequence analysis

To identify putative *IPT*, *CKX, ZOG* and *GLU* orthologue sequences in wheat, all the annotated family members of these multigene families from *Arabidopsis*, rice, maize and barley were used as query sequences to BLAST search the GenBank database (http://blast.ncbi.nlm.nih.gov). Subsequent verification utilised the recently released draft wheat genome (http://www.cerealsdb.uk.net/search_reads.htm). Isolation of these putative orthologues in wheat was carried out through direct sequencing of RT-PCR products using cDNA template derived from a mixed tissue sample containing several stages of developing leaves, spikes, carpels and seeds. PCR primers specific to each family member were designed based on the obtained sequence information. For family member (s) without sequence information in the available databases, degenerate primers were designed within the conserved regions of the same family member in barley, rice, maize and other monocot species, using Primer Premier 5.0 (Additional file 1).

PCR amplification was conducted in a 20 μl reaction mix containing 2 mM MgCl_2_, 10 pmol of each forward and reverse primer, 2 μl of 10-fold diluted cDNA and 1 U FastStart Taq polymerase (Roche), using cycling conditions as described in Song *et al.*[52]. The PCR products were separated on a 1% agarose gel. All bands of approximately the expected size were purified and sequenced on an ABS 3730 sequencer (Applied Bioscience) using the same primers as for the PCR. The raw sequences from at least two forward and two reverse directions were aligned using ClustalX software to correct the sequencing errors and to generate a consensus sequence for each gene of interest (GOI). The sequences were verified by BLAST searching the GenBank database. Full length or 3′ end cDNA sequences of selected genes were generated using gene specific primers and the GeneRacer^TM^ Kit or 3′ Race Kit (Invitrogen) following the manufacturer’s protocol. For phylogenetic analysis, orthologues from *Arabidopsis*, maize, rice and other closely related monocot species for each GOI were used to construct both a Neighbor-Joining (NJ) phylogentic tree using ClustalX software [53] and a Maximum Likelihood (ML) tree using PhyML3.0 software [54,55], with 1000 bootstrap replicates. Each tree was rooted with an out group orthologue*.* The nucleotide sequences reported in this paper have been submitted to the GenBank under the accession numbers listed in Table 1.

**Table 1:** Sequence similarity of the genes of interest (GOIs) isolated in the present study with their othologues

**GOI in this study**		**Orthologues in closely related species**			**Similarity (%)**	
**Gene name**	**Accession No.**	**Gene name**	Accession No.*	**Species**	**Protein**	**Nucleotide**
*TaIPT2*	JN128577	*ZmIPT2*	ABY78882	*Zea mays*	76.8	79.9
*TaIPT3*	JN128578	*OsIPT8*	AB239805	*Oryza sativa*	84.9	85.5
*TaIPT5*	JN128579	*ZmIPT5*	NP001121194	*Zea mays*	78.1	82.0
*TaIPT6*	JN128580	*ZmIPT6*	ABY78885	*Zea mays*	80.4	83.9
*TaIPT7*	JN128581	*ZmIPT7*	ABY78886	*Zea mays*	79.8	82.0
*TaIPT8*	JN128582	*ZmIPT8*	ABY78887	*Zea mays*	80.4	82.8
*TaCKX1*	JN128583	*ZmCKX1*	NP001105591	*Zea mays*	75.1	84.4
*TaCKX2*	JN128584	*ZmCKX5*	NP001185958	*Zea mays*	68.0	78.0
*TaCKX3*	JN128585	*ZmCKX10*	NP001146838	*Zea mays*	79.2	84.9
*TaCKX4*	JN128586	*OsCKX4*	NP001045353	*Oryza sativa*	88.9	76.1
*TaCKX6*	JN128587	*OsCKX3*	NP001064886.1	*Oryza sativa*	88.3	76.1
*TaCKX7*	JN128588	*OsCKX6*	Q6YW51	*Oryza sativa*	72.7	75.6
*TaCKX8*	JN128589	*ZmCKX8*	NP001185809	*Zea mays*	73.5	76.3
*TaCKX9*	JN128590	*OsCKX10*	Q5Z620	*Oryza sativa*	72.9	71.9
*TaCKX10*	JN128591	*OsCKX9*	Q75K78	*Oryza sativa*	86.9	72.4
*TaCKX11*	JN128592	*OsCKX8*	A2XVN3	*Oryza sativa*	81.5	74.6
*TacZOG1*	JN128593	*ZmcZOG1*	NP001105017	*Zea mays*	75.5	82.0
*TacZOG2-1*	JN128594	*ZmcZOG2*	Q8RXA5	*Zea mays*	75.4	82.6
*TacZOG2-2*	JN128595	*ZmcZOG2*	Q8RXA5	*Zea mays*	75.2	80.3
*TaZOG1*	JN128596	*ZmZOG3*	NO001148195	*Zea mays*	74.9	82.2
*TaZOG2*	JN128597	*ZmcZOG2*	NP001148091	*Zea mays*	64.1	75.6
*TaZOG3*	JN128598	*ZmZOG3*	NO001148195	*Zea mays*	72.3	80.7
*TaGLU1-1*	JN128599	*ZmGLU2*	ACG24271	*Zea mays*	67.9	73.3
*TaGLU1-2*	JN128600	*ZmGLU2*	ACG24271	*Zea mays*	67.3	73.1
*TaGLU1-3*	JN128601	*ZmGLU2*	ACG24271	*Zea mays*	68.0	74.1
*TaGLU2*	JN128602	*ZmGLU1*	NM001111984	*Zea mays*	95.2	94.9
*TaGLU3*	JN128603	*OsGLU25*	Q0DA21	*Oryza sativa*	85.8	NV
*TaGLU4*	JN128604	*HvGLU*	ACF07988	*Hordeum vulgare*	94.8	93.6
*TaRR1*	JN128605	*OsRR1*	CAI79405	*Oryza sativa*	73.7	79.7
*TaRR4*	JN128606	*OsRR4*	NP001045420	*Oryza sativa*	90.0	84.9
*TaRR5*	JN128607	*OsRR4*	NP001053348	*Oryza sativa*	95.0	89.4
*TaRR9*	JN128608	*OsRR9*	NP001065722	*Oryza sativa*	82.4	82.6

* If accessions numbers are different for protein and nucleotide sequences of the same gene, those for protein sequences are listed. NV, corresponding sequence not available.

### Quantitative expression of cytokinin synthesis and metabolism genes using qRT-PCR

Due to the hexaploid nature of bread wheat, in which each gene is present in triplicate sets located on homologous chromosomes of genomes A, B and D [42], gene expression studies involving large numbers of genes, particularly those in multigene families, is very challenging. Since, in general, the three homoeologues of the same locus share high sequence similarity [56], we employed a novel strategy to minimize the work load and to handle, simultaneously, as many family members as possible. PCR primers specific to the family member but which can be shared by the three homoeologues of the same gene were designed and used for quantitative expression studies using qRT-PCR (Additional file 2). The temporal expression of each putative triplicate gene set and of selected reference genes was quantified using an Mx3000P® real-time PCR instrument (Stratagene) and a home-made SYBR Green master mix or the KAPA SYBR® FAST qPCR Kits (Kapa Biosystems, Boston, USA). PCR amplification efficiency of each GOI and reference genes were determined as described in Song *et al.*[57]. At least two technical replicates for each of two biological replicates were carried out for samples from each developmental stage

Because the changes in endogenous cytokinins immediately post anthesis are well recorded [19-22], we thought to use type A response regulators as a surrogate indicator of endogenous cytokinin levels. These genes have recently been used as markers to track the *in planta* bioactive cytokinin concentration because their expression levels are reported to be proportionally correlated with the endogenous cytokinin content of the tissue [58,59].

Multiple reference genes, *GAPDH, β-actin*, *18 S rRNA*, and protein phosphatase gene (*PP2A*), were used as internal controls. For each reference gene, a correction factor (CF) for each cDNA sample was calculated using the Ct number of this cDNA sample divided by the average Ct number of all cDNA samples included in the same experiment. The values of three to four technical replicates were averaged to form the CF for each biological replicate of each reference gene. The final CF value for each biological replicate was created by averaging the CF values of the four reference genes. For each cDNA sample, the Ct number of each target gene was corrected before statistical analysis of its expression level.

### Data analysis

Experimental data were analysed as a randomised block experiment by analysis of variance as described in Song *et al.*[52], where PCR-run was the block factor, the stage of development was the treatment factor, and relative transcript abundance was the response variable.

## Results

### Isolation of cytokinin synthesis and metabolism genes

After using all of the annotated family members of *IPT*, *CKX, ZOG* and *GLU* in *Arabidopsis*, rice, maize and barley as query sequences to BLAST search the publically available genomic databases, we identified a large number of putative *IPT*, *CKX, ZOG* and *GLU* orthologue sequences of these multigene families in wheat. Most of the sequences were ESTs from GenBank and genomic DNA fragments from the recently released draft wheat genome (http://www.cerealsdb.uk.net/search_reads.htm). After manual assembly of the genomic fragments, all putative sequences were multiple aligned and phylogenetically analysed to determine the members of each gene family.

**Figure 1:**
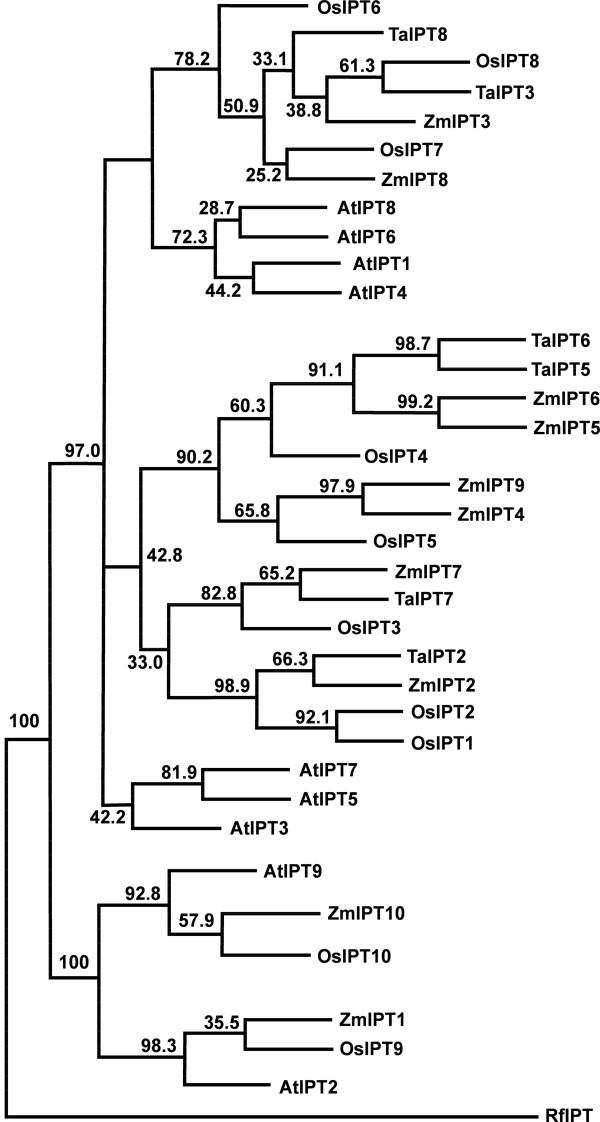
**The maximum likelihood (ML) phylogenetic tree for IPT proteins in*Arabidopsis thaliana*(AtIPT),*Oryza sativa*(OsIPT)*, Triticum aestivum*(TaIPT), and*Zea mays*(ZmIPT).** The tree was rooted using IPT protein from *Rhodococcus fascians* (RfIPT). Node values are percentages of bootstraps generated with 1000 bootstrap replicates.

**Figure 2:**
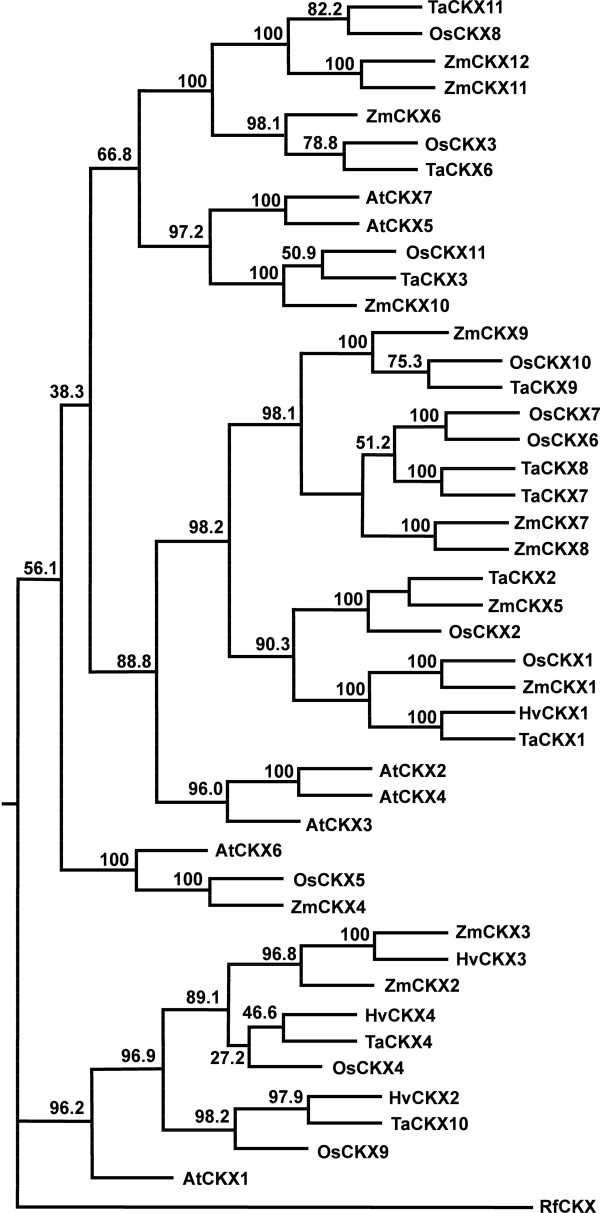
**The maximum likelihood (ML) phylogenetic tree for CKX proteins in*Arabidopsis thaliana*(AtCKX),*Oryza sativa*(OsCKX)*, Triticum aestivum*(TaCKX), and*Zea mays*(ZmCKX).** The tree was rooted using CKX protein from *Rhodococcus fascians* (RfCKX). Node values are percentages of bootstraps generated with 1000 bootstrap replicates.

**Figure 3:**
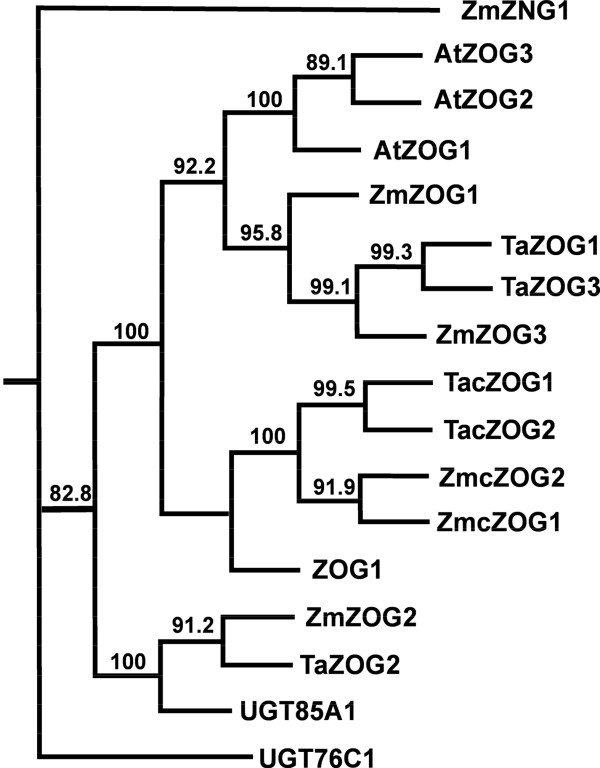
**The maximum likelihood (ML) phylogenetic tree for ZOG proteins in*Arabidopsis thaliana*(AtZOG, UGT),*Triticum aestivum*(TaZOG, TacZOG),*Zea mays*(ZmZOG, ZmcZOG) and*Phaseolus lunatus*(ZOG1).** The tree was rooted using cytokinin-N-glucosyltransferase 1 protein from *Zea mays* (ZmZNG1). Node values are percentages of bootstraps generated with 1000 bootstrap replicates.

**Figure 4:**
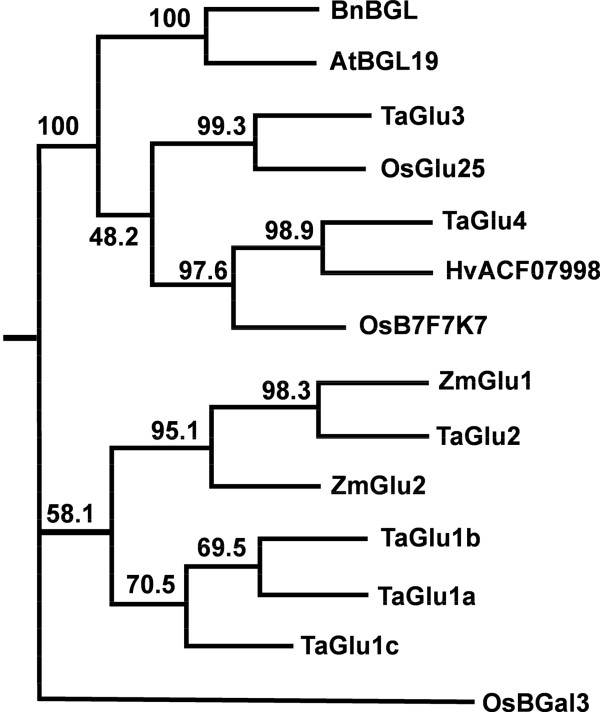
**The maximum likelihood (ML) phylogenetic tree for representative GLU proteins.** At, *Arabidopsis thaliana*; Os, *Oryza sativa*; Ta, *Triticum aestivum*; Zm, *Zea mays.* The tree was rooted using β-galactosidase 3 protein from *Oryza sativa* (OsBGal3). Node values are percentages of bootstraps generated with 1000 bootstrap replicates.

For the majority of members of these multigene families, three groups of sequences with very high similarity were detected. They were considered to be the three homoeologues of a putative gene. At least one set of specific PCR primers was designed for RT-PCR isolation of this gene. In total, we have isolated at least one homoeologous sequence from six *IPTs*, ten *CKXs*, five *ZOGs* and four *GLUs* (Table 1). Phylogenetic analysis (both ML and NJ trees) grouped each gene family into several distinct clusters consisting of gene sequences from both dicot and monocot species, with high bootstrap confidences between monocot and dicot sub-clusters. The bootstrap confidence levels among clusters were also high for *IPT*, *ZOG* and *GLU* genes while those for *CKX* genes were low (Figures 1, 2, 3, 4. Additional files 3, 4, 5, 6). Multiple sequence alignment results showed that *CKX* genes from different groups shared much greater sequence similarities than other gene families (Additional files 7, 8, 9, 10). Phylogenetic analysis also showed that these newly isolated sequences represented most of the family members in this species as predicted according to other closely related monocot species for which whole genome sequences were available, such as rice and maize (Figures 1, 2, 3, 4. Additional files 3, 4, 5, 6).

For the *IPT* gene family, *TaIPT2*, *3*, *5*, *6*, *7* and *8* were similar, respectively, to *ZmIPT2*, *3, 5*, *6*, *7* and *8* in maize and were designated *TaIPT2*, 3, *5*, *6*, *7* and *8,* respectively (Table 1, Figure 1, Additional files 3 and 7). *TaIPT2* is a 1012 bp fragment, with deduced protein sequence of 284 aa from the start point of the coding region and 160 bp of the 5′ UTR sequence. *TaIPT 5* and *6* fragments are 1561 and 1552 bp, respectively, with deduced protein sequences of 470 and 433 aa, respectively, from the start point of the coding region, and 151 and 253 bp of the 5′ UTR sequences, respectively. *TaIPT3*, *7* and *8* are 694, 753 and 950 bp fragments within the coding region of the genes.

For the *CKX* gene family, three sets of *TaCKXs* were almost identical to the recently annotated wheat *TaCKX1, 2,* and *3*[47-49] and were named accordingly. These were putative orthologues of *ZmCKX15* and *10*, respectively, in maize. Other putative *TaCKXs* grouped together with *ZmCKX4, 6-11*, and were designated *TaCKX4, 6-11*, respectively (Table 1, Figure 2, Additional files 4 and 8). *TaCKX12367* and *8* are cDNA fragments of 1500–2000 bp covering the whole coding region and contain fragments of both the 3′ and 5′ UTR sequences. *TaCKX4* and *9* fragments are 989 and 788 bp, respectively, with deduced protein sequences of 199 and 252 aa, respectively, near the 3′ end of the coding region, and 392 and 32 bp of the 3′ UTR sequences, respectively. *TaCKX10* and *11* are 541 and 735 bp fragments within the coding region of the genes.

Three putative *ZOG* genes shared high sequence similarity with *ZmZOG1*, *ZmZOG2* and *ZmZOG3*, and were named *TaZOG1*, *TaZOG2* and *TaZOG3*, respectively. Two other putative *ZOG* genes were orthologues of *cisZOG1* and *cisZOG2* in maize, and designated *TacisZOG1* and *TacisZOG2* (Table 1, Figure 3, Additional files 5 and 9). *TaZOG1* and *3*, and *TacisZOG1* and *2-1* are cDNA fragments of around 1400 bp covering the whole coding region and contain short fragments of both the 3′ and 5′ UTR sequences. *TaZOG2* and *TacisZOG2-2* are 1100 and 963 bp, respectively, with deduced protein sequences of 366 and 322 aa, respectively, within the coding region of the genes.

In addition to the one triplicate set of *GLU* genes, *TaGLU1a, 1b* and *1c,* investigated by Sue *et al.*[50], we have isolated three other *GLU* genes, *TaGLU2, 3* and *4*, which shared high sequence similarity to *ZmGLU2* in maize, and *OsGLU25* and B7F7K7 genes in rice, respectively (Table 1, Figure 4, Additional files 6 and 10). *TaGLU1-11-21-3* (the equivalents of *TaGLU1a, 1b* and *1c* [50]), *TaGLU2* and *TaGLU4* are cDNA fragments of 946–1648 bp in length near the 3′ end of the coding region, with deduced protein sequences of 277–501 aa, and 110–361 bp of the 3′ UTR sequences. *TaGLU3* is a 1080 bp fragment at the 5′ end of the coding region.

Six type-A response regulators, *TaRR1, 3-6* and *9* were isolated. They were putative orthologues of *OsRR1, 3-6* and *9*, respectively (Table 1).

### Chlorophyll content during flag leaf development and senescence

**Figure 5:**
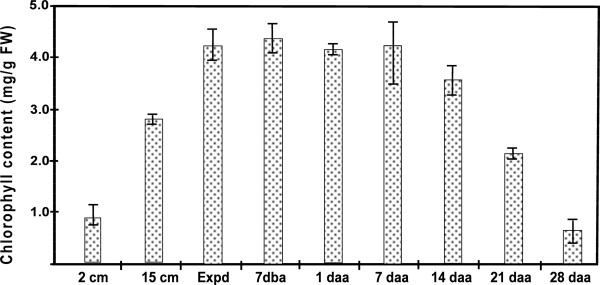
**Total chlorophyll content in flag leaf samples used for mRNA quantification of cytokinin regulatory genes in wheat.** Flag leaf sample order: 2 and 15 cm in length, expanded, days before and after anthesis.

The total chlorophyll content of the basal one-third of the flag leaves at different developmental stages was determined (Figure 5). The chlorophyll content in the flag leaf increased rapidly as the young leaf expanded to its full size, remained high (over 4 mg/g FW) over the next four weeks, from fully expanded to 7 daa, and then started to decrease. At 14 daa, when the end one-third of the leaf blade showed signs of senescence and the rest of the leaf remained green to the eye, the chlorophyll content in the basal one-third of the leaf had dropped to around 3.6 mg/g FW. At 21 daa, when the end two-fifths of the leaf were yellow in colour, about half of the chlorophyll in the basal one-third of the leaf was degraded, although visually the colour of this part of the leaf still remained green. At 28 daa, half of the leaf blade was yellow and the end one third had started to dry out. At that time, over 80% of the chlorophyll was degraded in the basal one-third of the leaf blade, where significant signs of senescence were now observable.

### Quantitative expression of cytokinin synthesis and metabolism genes

**Figure 6:**
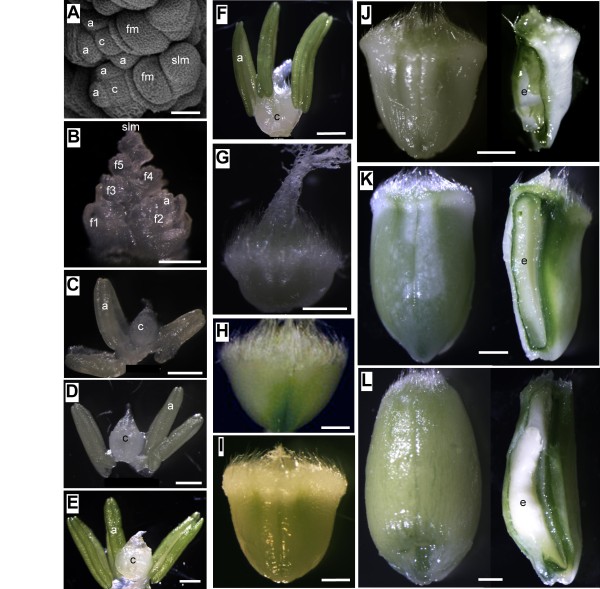
**Developmental stages of spike, carpel and seed samples used for mRNA quantification of cytokinin regulatory genes in wheat observedusing SEM (A) and light microscopy (B-L).A**, spike 0.5 cm in length; **B**, spike 2 cm in length; **C**, spike 10 cm in length; **D**, carpel 14 days before anthesis (dba); **E**, carpel 7 dba; **F**, carpel 1 dba; **G**, 1 day after anthesis (daa); **H**, 2 daa; **I**, 4 daa; **J**, 7 daa; **K**, 14 daa; **L**, 21 daa. a, anther; c, carpel; e, endosperm; f1-f5, florets 1–5 in a spikelet; fm, floral meristem; slm, spikelet meristem. Scale bars: A = 50 μm, B-E = 500 μm, F-K = 1 mm.

In order to determine the key genes with likely/critical roles during flag leaf, spike, carpel and early seed development, we first did a preliminary screen of all available *TaIPT*, *TaCKX*, *TaZOG* and *TaGLU* members utilizing cDNAs from root tips, elongating zone of internodes, leaves and reproductive tissues of mixed developmental stages. Gene specific PCR primers targeted to the three homoeologues of the same locus were used for qRT-PCR. Only those family members which expressed exclusively or mainly in leaves and reproductive tissues were then selected for further detailed expression studies across an extended series of spike, carpel, seed and flag leaf developmental stages. SEM and light microscopy were used to record the precise spike, carpel and seed developmental stages of the samples (Figure 6).

**Figure 7:**
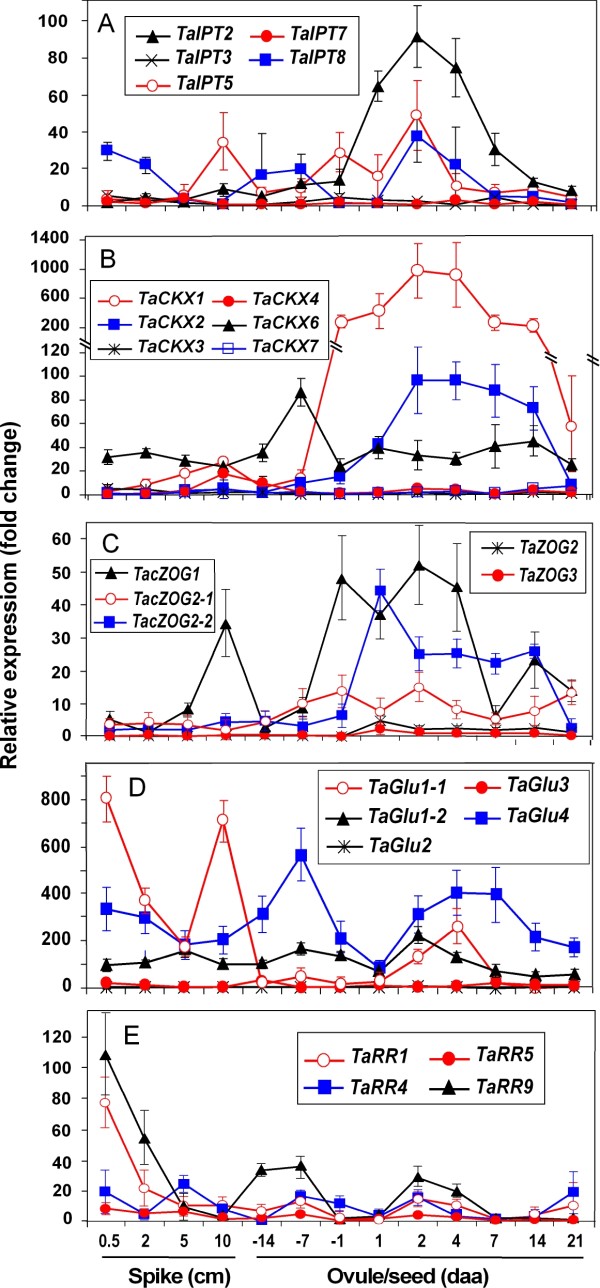
**Quantitative expression profiles of selected putative cytokinin regulatory genes during carpel and seed development in bread wheatA**, *TaIPT* genes; **B**, *TaCKX* genes; **C**, *TaZOG* genes; **D**, *TaGLU* genes; **E**, *TaRR* genes. Data values are means of relative mRNA levels in fold changes detected using qRT-PCR, using at least two technical replicates for each of the two biological replicates. Error bars represent the SD calculated for the combined technical and biological replicates. *GAPDH, β-actin*, *18 S rRNA*, and protein phosphatase gene (*PP2A*), were used as internal controls. Before quantification of the expression level of each of the target genes, the Ct numbers for each target gene were corrected by using the average correction factor determined for each of the four reference genes.

**Figure 8:**
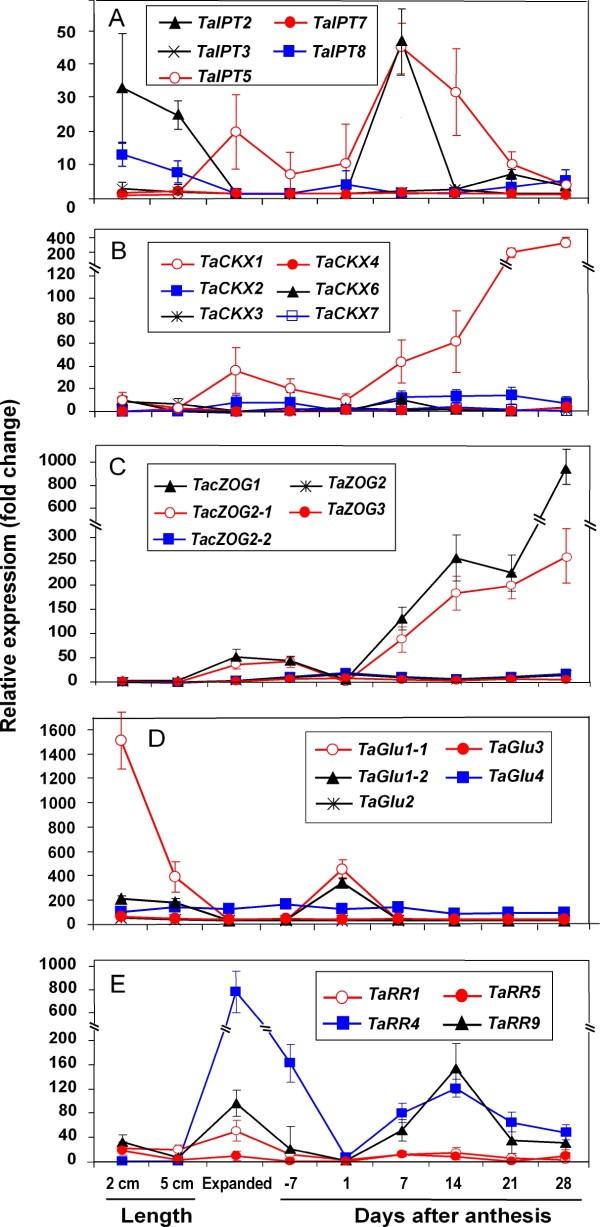
**Quantitative expression profiles of selected putative cytokinin regulatory genes during flag leaf development in bread wheat.** See Figure 7 for legend.

Using *GAPDH, β-actin*, *18 S rRNA*, and *PP2A* as reference genes, marked changes in the expression of members of all four gene families and of several type-A response regulators were observed during reproductive and flag leaf development (Figures 7 and 8).

During reproductive development, *TaIPT2* was highly expressed during early seed development (Figure 7A). Its relative expression level was low during early development of the spike and carpels but increased dramatically 1 to 4 daa, being 60- to 90-fold higher than the baseline expression, and 5 to 7-fold higher than that at 1 day before anthesis (dba). The mRNA abundance of *TaIPT2* dropped sharply 7 daa and returned to near the baseline at 21 daa. Both *TaIPT5* and *TaIPT8* showed three peaks of expression during reproductive development. One peak was shared by both genes and occurred around 2 daa, but with much less mRNA abundance than that of *TaIPT2*. While the other two expression peaks of *TaIPT5* appeared in 10 cm spikes and mature ovules at 1 dba, respectively, those for *TaIPT8* appeared in young spikes of less than 2 cm in length and mature carpels at 7-14 dba. The expression of *TaIPT* genes was, in general, lower during leaf development than reproductive development. While an expression peak appeared in the young leaf for *TaIPT2* and in the expanded leaf for *TaIPT5*, the two genes shared another expression peak at 7 daa. In comparison with reproductive development, the expression of *TaIPT8* was very low during all leaf development stages. The *TaIPT3* and *TaIPT7* genes expressed at very low levels in all samples (Figure 8A).

For the *TaCKX* gene family, *TaCKX1* was the most highly expressed family member, with expression from before anthesis through to14 daa and relative expression levels of 220 to 980-fold higher than that in the lowest expressed sample (Figure 7B). The expression pattern of *TaCKX2* was similar to that of *TaCKX1*, apart from the much lower expression level and the postponed expression window. While *TaCKX6* expressed during all stages of reproductive development, only one expression peak was detected at 7dba. In leaf samples, *TaCKX1* was the only family member with high expression, particularly in mature and senescing leaf tissue starting around 14 daa, when the chlorophyll content in the basal one-third just started to decrease. *TaCKX2* expressed constantly across all leaf samples but at a relatively low level (Figure 8B). Expression of the other *CKX* genes, i.e. *TaCKX3*, *4* and *7*, remained very low during both reproductive and leaf development, although slightly higher mRNA levels were detected in 10 cm spikes and young leaves for *TaCKX4*, and *TaCKX3*, respectively (Figures 7B, 8B).

In comparison with the senescing flag leaf, zeatin *O*-glucosyltransferase (*ZOG*) genes expressed at relatively low levels during reproductive development (Figure 7C). However, expression of *TacisZOG1* showed a peak of activity during spike development, as well as expression from 1 dba and throughout early grain development. Additionally, *TacisZOG2-2* expressed from 1 to 14 daa. Both *TacisZOG1 and TacisZOG2-1* expressed during leaf expansion and in the mature leaf until anthesis. In older leaves, while other *ZOG* genes expressed at very low levels, there was a sharp increase of mRNA for both *TacisZOG1* and *TacisZOG2-1*, The expression of *TacisZOG1* was over 900-fold higher than that in the lowest expressed sample tested and was particularly high from 21 daa, when the chlorophyll content in the basal one-third of the leaf started to decrease. Expression of one homoeologue of *TacisZOG2*, *TacisZOG2-1* mimicked the trend of *TacisZOG1* in leaves, whereas the *TacisZOG2-2* homoeologue showed virtually no expression (Figure 8C). In contrast, in the developing ovule and young seed, while the *TacisZOG2-2* homoeologue was showing a strong pattern of expression, the *TacisZOG2-1* showed only low, fluctuating expression.

For the *β*-glucosidase (*GLU*) gene family, an extremely high expression of *TaGLU1-1*, with a relative expression level over 1400-fold, was detected in young expanding leaves 2 cm in length (Figure 7D). Multiple expression peaks of the same gene, with relative expression over 200- to 800-fold, were also detected in 0.5 and 10 cm spikes, and in leaves at anthesis. In contrast, another allele from the same homoeologue gene, *TaGLU1-2*, expressed consistently during spike, seed and leaf development, with the exception of in leaves at anthesis, during which the relative expression level was 300-fold higher than the baseline and similar to *TaGLU1-1*. The highest expression of *TaGLU4* was detected at 7 dba, presenting an over 500-fold up regulation. *TaGLU4* also expressed at a relatively high level at most other stages of reproductive development, but at a consistently lower level in most leaf samples. Expression of other *GLU* family members, such as *TaGLU2* and *TaGLU3*, was low across all tested samples, without any obvious expression patterns during reproductive and leaf development (Figure 8D).

With the exception at the time of anthesis, elevated expression of several response regulators (*RR*) was detected in fully expanded and mature leaves (Figure 8E). This expression was coincident with that of *TaIPT* gene expression in the leaves. Although several RR members did express at relatively high levels in the early stages of spike development (Figure 7E), there was only a relatively low level of RR expression during reproductive development when *TaIPT* gene family members were expressing. However, the small peak of *TaRR9* is coincident with the peak of *IPT* expression at 2 daa.

## Discussion

Phylogenetic analysis showed that *TaCKX1* shares high sequence similarity with orthologues from other monocot species, including *HvCKX1* in barley, *ZmCKX1* in maize and *OsCKX1* in rice (Figure 2), suggesting that comparative phylogenetic analysis based on sequence similarity is a powerful method to identify homologous genes and to predict their physiological functions in target species. Identification of orthologues across species is essential to predict gene function in newly sequenced genomes such as wheat for which a draft genome has recently been released (http://www.cerealsdb.uk.net/search_reads.htm). Indeed, when Gu *et al.*[34] combined a phylogenetic analysis with comparative expression of *Arabidopsis*, poplar, maize and rice *CKX*s in various tissues, they observed a general association of sequence similarity with expression patterns. Phylogenetic analysis has been widely used to classify gene families and predict their functional orthologues, and has been successfully used to generate functional categories within large gene families such as the WRKY and bHLH transcription factor families [60,61]. However, phylogenetic analysis does not allow the differential expression of homoeologous genes to be determined.

In this study, we isolated most members of the major multi-gene families, *TaIPTs*, *TaCKXs*, *TaZOGs*, and *TaGLUs* involved in cytokinin synthesis and metabolism. Potential functional homoeologues from the three genomes, A, B and D, were pre-screened based on their expression in selected tissue types and in developmental stages closely related to grain yield determination. By using a novel strategy with specific PCR primers shared among the three homoeologues of each family member, we have been able to reveal detailed expression profiles of family members of the four multigene families during leaf, spike and seed development using qRT-PCR. Such information is required before determining which are the key genes and homoeologues contributing to cytokinin homeostasis within specific organs and developmental stages. These key genes then become the target for further investigation and genetic manipulation toward yield improvement in a breeding programme.

In plants, the rate limiting enzyme in cytokinin biosynthesis is considered to be IPT, which functions by attaching the isopentenyl side chain to the N^6^ moiety of ADP or ATP. At least three of the six *IPT* genes tested in this study, *TaIPT2TaIPT5* and *TaIPT8* showed specific expressed patterns during spike, carpel, seed and leaf development. All three genes were expressed at the highest levels at early seed development immediately after anthesis (Figure 7A), a developmental window coincident with the cell division period, during which the cell number of the endosperm is determined, and during which endogenous cytokinin levels peak [e.g. [19-21]. Expression of *IPT* family members was also detected in young spikes and developing carpels, suggesting that these genes should be further investigated as players in determining the number as well as the size of the reproductive organs. Interestingly, these *TaIPT* genes expressed at relatively low levels during the expansion and the fully expanded phase of the leaf. Together with similar results recently reported in maize [32,33], this suggests that it would be possible to manipulate *IPT* activity during reproductive development without significantly affecting normal leaf functions. An initial target for enhanced expression could be *TaIPT8*, which does not express highly in leaves, thereby avoiding creating competition between sink and source.

The *CKX* gene family members have been shown to express in different plant tissues and to play essential roles in controlling cytokinin levels during plant growth and development [62]. Information about the expression and physiological function of *TaCKXs* was only recently available and only for *TaCKX1, 2* and *3*[47-49]. While investigating the expression of ten sets of *TaCKX* genes, we showed that at least three genes are specifically or preferentially expressed during seed development (Figure 7B). The strong and specific expression of the *TaCKX1* gene during early seed development provides a useful candidate target. By perturbing cytokinin homeostasis during this key yield determining stage, i.e. by down regulating the expression of any one of *CKX1, 2* or *6* during seed development and disturbing the normal mechanism of cytokinin homeostasis, the resulting elevated cytokinin level may lead to enhanced seed number and size [8]. Indeed, Zalewski *et al.*[13] were able to increase grain yield of barley by down regulating the *TaCKX1* orthologue, *HvCKX1*, using RNAi.

*O*-glucosylation is a major step in the metabolism of cytokinins. Since *O*-glucosylation is reversible and *O*-glucosides are resistant to cleavage of the *N*^6^-side chain by CKX, this conversion forms part of the homeostatic control of active cytokinin levels through temporary storage of cytokinins [11]. Interestingly, *TacisZOG1* showed elevated expression pre-anthesis (Figure 3), at a time when endogenous *O*-glucosides start to accumulate in the developing ovule [19]. Extremely high level of expression was detected for two *cis-ZOG* genes, *TacisZOG1* and *TacisZOG2-1*. The dramatic increase of *TacisZOG* expression during leaf senescence in the present study agrees with early observations of high levels of *O*-glucosides in senescing leaves [63] and cytokinin inactivation by *O*-glucosylation promoted by senescence-related processes in petunia [64]. In contrast, Vyroubalová *et al.*[33] only detected low expression of *ZmcisZOG* in old leaves of maize. This difference might be due to differences in cytokinin metabolism during senescence in these species [63].

The *β*d-glucosidases are members of the GH1 family, which is implicated in the hydrolysis of a number of plant secondary metabolites, including the regulation of the biological activity of cytokinins [65]. Until now, Zm-p60/*ZmGLU1* in maize, *TaGLU1* in wheat and Bgl4 in *Brassica napus* are the only enzymes for which the ability to release cytokinins from *O*-glucoside conjugation has been demonstrated [50,65-67]. In wheat, only one homoeologous set of *GLU* genes, *TaGLU1a, b* and *c,* has been investigated [50], but with no information shown on *in planta* expression during reproductive and leaf development. Our qRT-PCR data showed high expression of *TaGLU1-1*, the equivalent of *TaGLU1a* in Sue et al. [50], in 0.5 cm spike, 10 cm spike and 2 cm leaf. Using a *ZmGLU1P-GUS* construct, Gu *et al.*[68] observed a significant up-regulation of the maize *β*-glucosidase gene, *ZmGLU1,* in immature transgenic tobacco seeds. Vyroubalová *et al.*[33] also detected high expression of *ZmGLU* in immature ears and young leaves of maize. Therefore, we speculated that this gene may play an important role in reproductive organ initiation and seed development.

Interestingly, and in agreement with the pattern shown in our preliminary study [41], at anthesis, when expression of *IPTCKX* and *ZOG* genes was uniformly low in the flag leaf, the *TaGLU1* gene showed a marked peak of expression, with over a 300-fold up-regulation. From anthesis, the flag leaf provides significant resources to the developing seed. We postulate that the high β-glucosidase gene activity at this stage could lead to the release of cytokinin from conjugation in the leaf and provide a source of cytokinin for the developing carpels/seeds. Clearly, experiments to support this supposition still need to be performed.

The modest correlation of response regulators with *TaIPT* gene expression during the early stage of seed development when endogenous cytokinin levels are substantially elevated [19-21] indicates that response regulators may not be good surrogates of endogenous cytokinin content. However, the elevated expression of members of all four gene families at 2 daa is indicative of dynamic turnover of cytokinins at this key stage of seed development.

An interesting trend shown by our data, and that of our preliminary study [41], is that several of the highly expressed genes in the seeds have low expression in the leaves and *vice versa*, further suggesting that expression of individual family members of the cytokinin synthesis and metabolism genes are tissue specific. As a monocarpic plant, wheat development is characterized by a rapid translocation of metabolites from leaves to developing grains after anthesis. Consequently, delayed leaf senescence at a late stage of seed development may not be beneficial to the final grain yield due to the decreased nutrient translocation from leaves to seeds as suggested by Sýkorová *et al.*[69]. The independent and variable expression profiles of different members within or among cytokinin regulatory gene families provides useful flexibility for independently manipulating the endogenous bioactive cytokinin levels in reproductive and vegetative tissues to achieve maximal seed yield.

The expression levels shown in this study represent at least one of the three homoeologues for each locus in all cases apart from the *TaZOG2-1TaZOG2-2TaGLU1-1* and *TaGLU1-2* genes, in which the three homoeologous genes showed different expression profiles. The third homoeologue in these cases displayed little activity in the initial screen for highly expressed genes. The expression among homoeologous genes of other cytokinin regulatory genes may also vary, or individual homoeologous genes could even be silenced in some or all of the tissue samples analysed. Homoeologous gene silencing and/or differential expression is a phenomenon widely documented in bread wheat [43-46] and other polyploid species including tetraploid cotton [70], and tetraploid *Tragopogon mirus*[71].

Given that homoeologous expression can be differentially regulated by genetic and epigenetic mechanisms in a tissue and developmentally-specific manner [43-45], detection and pyramiding of interesting homoeologues for improvement of target traits has recently been used in wheat breeding programs [72]. For instance, in an attempt to improve the bread-making quality of hexaploid wheat, homoeologous recombination has been used to increase the copy number of the stronger flour quality contributing homoeologue, *Glu-D1*, of the HMW-GS gene. A partially isohomoeoallelic line, in which the chromosome 1A homoeologue was replaced by the chromosome 1D homoeologue, showed significantly improved bread making quality. The improved quality can be explained by the duplication of the *Glu-D1* homoeologue [73,74]. Using a different approach, TILLING in combination with homoeologue-specific primers, Slade *et al.*[75] obtained a triple homozygote loss-of-function of the *Wx* gene and obtained a line with waxy phenotype and improved grain quality,

Therefore, from a breeding perspective, the above strategy, of accumulating targeted homoeologues, could also be applied to the cytokinin synthesis and metabolic gene families. To improve seed yield, higher endogenous cytokinin levels could be achieved through tissue-specific increased expression of multiple homoeologues of the *IPT* genes *TaIPT2*, *TaIPT5* and *TaIPT8*, or through decreased homoeologue expression of *CKX* genes *TaCKX1* and *TaCKX2*. This could be achieved using a number of genetic tools, such as over-expression, down-regulation, TILLING and MAS strategies. However, while the differential expression profile of each homoeologue elucidated in this study could be directly used for genetic manipulation towards the improvement of the grain yield, precise genome allocation of these positive homoeologues is important for further understanding the genetic control, and for facilitating the efficient accumulation, of these homoeologues within the same locus and across different loci.

## Conclusions

We have demonstrated that the expression patterns of individual members of the *TaIPTs*, *TaCKXs*, *TaZOGs*, and *TaGLUs* multigene families were tissue and developmentally-specific during spike, ovule, seed and flag leaf development. This supports the suggestions that the four multigene families play important roles in maintaining cytokinin homeostasis during reproductive development and will contribute to grain yield and quality. We suggest that artificial disturbance of endogenous cytokinin levels at specific stages of organ development through targeted manipulation of particular family members is a practical possibility, and have identified several key candidate genes for genetic improvement of grain yield in this species.

## Competing interest

The authors declare that they have no competing interests.

## Authors’ contributions

PEJ and JS conceived and designed the experiments. PEJ was the PI of the project, supervised all the work, contributed to and edited the manuscript. JS performed the experiments, analyzed the data and drafted the manuscript. LJ provided assistance in performing the experiments and preparing the manuscript. All authors read and approved the final manuscript.

## Supplementary Material

Additional file 1 PCR primers used for cytokinin regulatory gene isolation in bread wheat. Click here for file

Additional file 2 Specific PCR primers for expression analysis using qRT-PCR. Click here for file

Additional file 3 **Neighbor Joining phylogenetic tree for IPT proteins in*Arabidopsis thaliana***, ***Oryza sativa, Triticum aestivum***, **and*Zea mays*.** Click here for file

Additional file 4 **Neighbor Joining phylogenetic tree for CKX proteins in*Arabidopsis thaliana***, ***Oryza sativa, Triticum aestivum***, **and*Zea mays*.** Click here for file

Additional file 5 **Neighbor Joining phylogenetic tree for ZOG proteins in*Arabidopsis thaliana***, ***Oryza sativa, Triticum aestivum***, **and*Zea mays*.** Click here for file

Additional file 6 Neighbor Joining phylogenetic tree for GLU proteins in wheat and representative species. Click here for file

Additional file 7 **Comparison of deduced protein sequences of selected*TaIPT*gene fragments with representative IPT proteins in maize and rice.** Click here for file

Additional file 8 **Comparison of deduced protein sequences of selected*TaCKX*gene fragments with representative CKX proteins in maize and rice.** Click here for file

Additional file 9 **Comparison of deduced protein sequence of TaZOG and their orthologues in selected protein sequences in maize and*Phaseolus lunatus.*** Click here for file

Additional file 10 **Comparison of deduced protein sequence of*TaGLU*genes and their orthologues in maize, rice, and barley.** Click here for file
