# Supplementary material for: Co-ordinate regulation of cytokinin gene family members during flag leaf and reproductive development in wheat
Source: BMC Plant Biol. 2012 Jun 6;12:78. doi: 10.1186/1471-2229-12-78 (PMC3410795; doi:10.1186/1471-2229-12-78)
Supplement: Additional file 1 — PCR primers used for cytokinin regulatory gene isolation in bread wheat. [file 1471-2229-12-78-S1.doc]

Additional file 1. PCR primers used for cytokinin regulatory gene isolation in bread wheat

| **Primer names** | **Sequences (5′ to 3′)** |
| --- | --- |
| IPT F1 | CAAGTCSAAGCTCGCCATCBSC |
| IPT R1 | TCCCASCCKTCGAGCGYGCT |
| IPT F2 | ACGGGGMTGGTGGTGATCGT |
| IPT R2 | AAGCTSCGGATGGYGGCGAG |
| IPT F3 | RCYGCTGGTCAGTGCCAACAA |
| IPT R3 | CCGSSGCGRGAGCRAACA |
| IPT F4 | CCAAGCACAAGGCCGTSGT |
| IPT R4 | GAAGGACCKSACSGCGTCRAT |
| IPT F5 | CAAGTCCAAGCTGGCCATCGA |
| IPT R5 | CGYGGAGCCGGTGGATCTTC |
| CKX F1 | TTCTTCACCGACGCCGACGT |
| CKX R1 | CAGCYTCTTGGGGTCGTACTTG |
| CKX F2 | ASATGGTGACGTGYTCSAAGGAGA |
| CKX R2 | GACCGMAGGATCCCMACCGTG |
| CKX F3 | GACGCGGAGTGGCTGGTGAC |
| CKX R3 | KCGGGAAGTAKRYCTTGWAGTCGTAG |
| CKX F4 | CCTYGARCTRACSAAGAACTTCAATAGT |
| CKX R4 | AATRCACTRCCCTCATGCAAACTTCTG |
| CKX F5 | YATMTCYMTGCTKCTYAGCTTCCTCTC |
| CKX R5 | STGGGAYGCYATGTGGCTCATC |
| CKX F6 | YBRCSRCCGCRTCAACGTGTC |
| CKX R6 | KHCTCRKYRTAGTACATGGCGSCTTC |
| CKX F7 | GGTAAGGTGGMTAMGAGTTCTCTACTT |
| CKX R7 | GAGCTAAGATGGCCAAGGGGTC |
| cisZOG F1 | MGGRAAYTCRATGGCGGTTG |
| cisZOG R1 | CSASGAACTCCYTGGACATGCAG |
| cisZOG F2 | CCAYCTGAACCAGCTSMTSCAC |
| cisZOG R2 | CCCAGAYGAAMCKCTGCYTGCTG |
| ZOG F1 | RRAGATGGCKGCGTCGGAG |
| ZOG R1 | GTCCYYSACGCGCKMCTCGA |
| ZOG F2 | CACGTSACGCCGATGMTGAAG |
| ZOG R2 | KCCCKGCCSTGGAGCCACT |
| ZOG F3 | MSGMGCCGCTSSAGGVGTAC |
| ZOG R3 | GGYGSGAVAGGATGGTCACCTG |
| Glu1 F | TCGCTCAGAGAGCCATACACTGC |
| Glu1a R | ACGGAAACGGAAGTAACTGTCACC |
| Glu1b R | ACAGAAACGGAAGCAGCTGTCATG |
| Glu1c R | ATAGGGAAACGGAAGCAGTCACGGCTGG |

| **Primer names** | **Sequences (5′ to 3′)** |
| --- | --- |
| Glu F1 | CKAGCYCAYGCTGAGGCTGTTG |
| Glu R1 | GCGRTCGACGTAGACAATGCCA |
| Glu F2 | CGGYGCTGTTGCTGGTRGYACT |
| Glu R2 | RAGGAACCAKCCWAKGTGGAAGTC |
| RR F1 | GGGTGAYGAGGGTGCTGSTG |
| RR R1 | KWGAGGAAGCAGCCGTTGGAC |
| RR F2 | YKTCAACCAAATCAACAGGCATCA |
| RR R2 | GYYGCTACCGCATGGGCAT |
| RR F3 | CGCCATGACGGTGGTSGAC |
| RR R3 | GCCGCCTTCCKCTTYCTGCT |
| RR F4 | GCTGCTCCCRCSCAGGCTC |
| RR R4 | CGGTGGTTGMTGATCAYCKGAT |
| RR F5 | TCTCTCATGCCAAGCGCACA |
| RR R5 | TCYTTGCATCTGCTTTTCAKTATGTG |
