# Supplementary material for: Co-ordinate regulation of cytokinin gene family members during flag leaf and reproductive development in wheat
Source: BMC Plant Biol. 2012 Jun 6;12:78. doi: 10.1186/1471-2229-12-78 (PMC3410795; doi:10.1186/1471-2229-12-78)
Supplement: Additional file 2 — Specific PCR primers for expression analysis using qRT-PCR. [file 1471-2229-12-78-S2.doc]

Additional file 2. Specific PCR primers for expression analysis using qRT-PCR

| **Primer names** | **Sequences (5′ to 3′)** |
| --- | --- |
| TaIPT2 F | GCCGGGGATGATGGAGTGGTA |
| TaIPT2 R | GCTGGAGGACGAGGCGGAA |
| TaIPT3 F | TGGATGTCGACGAGGCAGTTCT |
| TaIPT3 R | ATGCGCCGGATCTTGCCGA |
| TaIPT5 F | CACAGACAAGCTGCACGAAGGA |
| TaIPT5 R | CGATCGGTCAGCTTGTGTACCAAC |
| TaIPT6 F | CAAGAAACAATCCGCACGAAGAA |
| TaIPT6 R | GATCGGTCAGCTTGTGTACGACAC |
| TaIPT7 F | GCGGTGCTGGAACGGTACGT |
| TaIPT7 R | TGACTGCACACCAGCCTGCA |
| TaIPT8 F | GACGTGGAGGAGGCGCTCCT |
| TaIPT8 R | ATGCGCCGGATCTTGGACA |
| TaCKX1 F: | GGAGGTGGCGCTGGACAAGATC |
| TaCKX1 R | GCAGAACCGCAGTATCTTCTGGT |
| TaCKX2 F | CCAGAGGAGGAGGAGGTGTTCTAC |
| TaCKX2 R | TTGGCCGGACCAAAGTGCTT |
| TaCKX3 F | GGAGGGCTTCGCGTTCGTG |
| TaCKX3 R | CAGGCCCCGCACGTACTTGA |
| TaCKX4 F | TGCTGTCTCGGCTGAGATACATACAG |
| TaCKX4 R | TGACGTCCTGTTGTCCCACTTTG |
| TaCKX6 F | CATCTCAGTGCAGATGAGCCACA |
| TaCKX6 R | TGCAGGCAGTGGTGGGAGCA |
| TaCKX7 F | GTGCGCGTGGAGGAGGTTGT |
| TaCKX7 R | CACCATGTAGAATACGTCCTCGACTG |
| TaCKX8 F | TGCGCGTGGAGGAGGCTGA |
| TaCKX8 R | ACAGTGTAGAATACGTCCTCGCCAG |
| TaCKX9 F | GGCGTCCTCAGGGACACCA |
| TaCKX9 R | CTCGCGGGTCGTACGTGGA |
| TaCKX10 F | GGTAAGGTGGATAAGAGTTCTCTACTT |
| TaCKX10 R | ATCTGAGTTGAGATAGTAGTGCATGGA |
| TaCKX11 F | AGCAACGTCCTGCAGCTCCAA |
| TaCKX11 R | GAGCTGCGGATGGAGTGCTCA |
| TacicZOG1 F | TGTTCAAGGACCAGAAGCTCTTTG |
| TacicZOG1 R | CAATACCCAGATGAACCTCTGCCT |
| TacicZOG2a F | CCTGCATGTCCAAGGAGTTCCTC |
| TacicZOG2a R | GAAGGACACGTAGAGCACCGACT |
| TacicZOG2b F | GACGGAGAAGGAGATGGAGGAACA |
| TacicZOG2b R | GAAGGACACGTAGAGCACCGATGA |

| **Primer names** | **Sequences (5′ to 3′)** |
| --- | --- |
| TaZOG1 F | CGAGGAGCGCGTCAAGGACA |
| TaZOG1 R | TCTCCACGTCGCCGCTAGTGA |
| TaZOG2 F | ACTCACCGAGCAGCTGGTCTCA |
| TaZOG2 R | CCCTTCACCAGGTCGTTCCTCA |
| TaZOG3 F | GAGTCGCGCGTGGAGGACA |
| TaZOG3 R | GCCTCCTCGGGGACGTTCTT |
| TaGlu1a F | AATAACAACGCAACGGTGACAGTTA |
| TaGlu1a R | GATCCCGACGACATGCAAACA |
| TaGlu1b F | TAACAACGCAACCATGACAGCTG |
| TaGlu1b R | CCGACGCCAAGCGAGCAT |
| TaGlu1c F | ACCAATAACAAGCCAGCCGTGAC |
| TaGlu1c R | CGACGAGTTCACAAGGAACACGA |
| TaGlu2 F | GAAGCTCGCCGGTTCCTATAACAT |
| TaGlu2 R | CGATTCCGTTCTCGGTGATGTAGATA |
| TaGlu3 F | GGAAAAGGTATACAACCATATGTAACAC |
| TaGlu3 R | GAAAAACACCAGCATGAGCTAAGA |
| TaGlu4 F | ACGTGACGGTCGACGAGTACCATA |
| TaGlu4 R | TCTCCAAACACCTTGAAGCAGAACT |
| TaRR1 F | GGCTACGACCTCCTCAAAGCCA |
| TaRR1 R | GCGGCACATCCTTGCTCTGA |
| TaRR3 F | ACGGTGGACAGCGGGAAGAAG |
| TaRR3 R | GATCCTGGACGGCGAGTTCTC |
| TaRR4 F | AGGAGGTGGGCGTGAATTTGA |
| TaRR4 R | TGCGACTTGAGCTTCTTCATGTCA |
| TaRR5 F | GTTCTCGCGGTGGACGACAG |
| TaRR5 R | TCCAAGCATCTGCTGATCCTTGT |
| TaRR6 F | GTGATGACCGACGCCGACGA |
| TaRR6R | GTCGCAGAGTCCACGGTGGTAAC |
| TaRR9F | CGTGGATGACAGCGTCCTTGA |
| TaRR9R | CTTCCTCCAGGCATCTGTTGATTC |
