# Supplementary material for: Co-ordinate regulation of cytokinin gene family members during flag leaf and reproductive development in wheat
Source: BMC Plant Biol. 2012 Jun 6;12:78. doi: 10.1186/1471-2229-12-78 (PMC3410795; doi:10.1186/1471-2229-12-78)
Supplement: Additional file 3 — Neighbor Joining phylogenetic tree for IPT proteins inArabidopsis thaliana, Oryza sativa, Triticum aestivum, andZea mays. [file 1471-2229-12-78-S3.doc]

Additional file 3. Neighbor Joining phylogenetic tree for IPT proteins in *Arabidopsis thaliana* (AtIPT), Oryza sativa (OsIPT), *Triticum aestivum* (TaIPT), and *Zea mays* (ZmIPT)

The tree was rooted using IPT protein from *Rhodococcus fascians* (RfIPT). Node values are the number of bootstraps for 1000 bootstrap replicates.
