# Supplementary material for: Co-ordinate regulation of cytokinin gene family members during flag leaf and reproductive development in wheat
Source: BMC Plant Biol. 2012 Jun 6;12:78. doi: 10.1186/1471-2229-12-78 (PMC3410795; doi:10.1186/1471-2229-12-78)
Supplement: Additional file 5 — Neighbor Joining phylogenetic tree for ZOG proteins inArabidopsis thaliana, Oryza sativa, Triticum aestivum, andZea mays. [file 1471-2229-12-78-S5.doc]

Additional file 5. Neighbor Joining phylogenetic tree for ZOG proteins in *Arabidopsis thaliana*, Oryza sativa, *Triticum aestivum*, and *Zea mays*

The tree was rooted using cytokinin-N-glucosyltransferase 1 protein from *Zea mays* (ZmZNG1). Node values are the number of bootstraps for 1000 bootstrap replicates.
