# Supplementary material for: Co-ordinate regulation of cytokinin gene family members during flag leaf and reproductive development in wheat
Source: BMC Plant Biol. 2012 Jun 6;12:78. doi: 10.1186/1471-2229-12-78 (PMC3410795; doi:10.1186/1471-2229-12-78)
Supplement: Additional file 6 — Neighbor Joining phylogenetic tree for GLU proteins in wheat and representative species. [file 1471-2229-12-78-S6.doc]

Additional file 6. Neighbor Joining phylogenetic tree for GLU proteins in wheat and representative species

At, *Arabidopsis thaliana*; Os, Oryza sativa; Ta, *Triticum aestivum*; Zm, *Zea mays.* The tree was rooted using β-galactosidase 3 protein from Oryza sativa (OsBGal3). Node values are the number of bootstraps for 1000 bootstrap replicates.
