# Supplementary material for: Co-ordinate regulation of cytokinin gene family members during flag leaf and reproductive development in wheat
Source: BMC Plant Biol. 2012 Jun 6;12:78. doi: 10.1186/1471-2229-12-78 (PMC3410795; doi:10.1186/1471-2229-12-78)
Supplement: Additional file 7 — Comparison of deduced protein sequences of selectedTaIPTgene fragments with representative IPT proteins in maize and rice. [file 1471-2229-12-78-S7.doc]

TaIPT2 (1) --------MEQ-RRGGKPKVVFVLGATATGKSKLAISLAKRFGGEVINSDKIQVYDGVPI

ZmIPT2 (1) --------MEHGAVAGKPKVVFVLGATATGKSKLAIALAERFNGEVINADKIQVHDGVPI

OsIPT2 (1) ---MEYHVGGVIGQSPKPKVVFVLGATATGKSKLAISIAERFGGEVINSDKIQVHDGFPI

TaIPT7 (1) -----------ASGNGKAKVVIVMGATATGKSKLAIDLALRFGGEVVNSDKIQVHDGLDV

ZmIPT7 (1) --MAGVNGATASGGDNKAKVVLVMGATATGKSKLAIDLALRFGGEVVNSDKIQVHDGLDV

TaIPT5 (26) --RPPPPLLVRH-AAAKHKAVVVMGATGTGKSRLAIDLALRFGGEVINSDKMQVYDGLDI

TaIPT6 (1) ------------------KAVVVMGATGTGKSRLAIDLALRFGGEVINSDKMQVYDGLDI

ZmIPT5 (35) -PPLSVGGACRR-VAAKHKAVVVLGATGTGKSRLAIDLALRFGGEVINSDKIQAHAGLDV

ZmIPT6 (33) -AAPLSVVGCRR-MAAKHKAVVVLGATGTGKSRLAIDLALRFGGEVINSDKIQAYAGLDV

OsIPT4 (36) -AVPVAPLVLRHGAGVKHKAVVVMGATGTGKSRLAVDLALRFGGEVINSDKMQIHSGLDV

ZmIPT4 (48) APPPPLVSANNRHAGAKHKAVVVMGATGTGKSRLAVDLALRFGGEVINSDKIQLHAGLDV

OsIPT5 (37) -PPPPLVSVS-RSMVAKHKAVVVMGATGTGKTRLAVDLALQFGGEVINADKLQLHRGLDV

TaIPT3 (1) ----------------DTRLVVIVGATGTGKTKLSIDAALELGGEVVNADKIQLYRGLDV

OsIPT8 (16) GRGGVASTTAVRP---STGMVVIVGATGTGKTKLSIDAAQELAGEVVNADKIQLYDGLDV

OsIPT7 (16) AAASRPLRLHRRPGGEDTRMVVIVGATGTGKTKLSIDAAKVIGGEVVNADKIQLYDGLDV

ZmIPT8 (61) SCSASSSSTPARPRG--TGMVVIVGATGTGKTKLSIDAAEAVGGEVVNADKIQLYAGLDV

TaIPT8 (24) RGGGGSGWGEGRPR-----LVVIVGATGTGKTKLSIDAARALGGEVVNADKIQLYQGLDV

OsIPT6 (10) CFLNMLGN----------KLVVIIGATGTGKTRLSIEIAKAIGGEVVNADKMQIYDGLDI

TaIPT2 (52) LTNKVTPEETAGVPHHLLGGVHPDA-DFTAEDFRREAAAAVARVLAAGRLPVVAGGSNTY

ZmIPT2 (53) ITNKVTEEEQGGVPHHLLSVRHPDA-DFTAEEFRREAASAVARVLSAGRLPVVAGGSNTY

OsIPT2 (58) ITNKVTEEERAGVPHHLLGVLHPDA-DFTAEDFRREAAAAVARVLAAGRLPVVAGGSNTY

TaIPT7 (50) VTNKATARERAGVPHHLIGGVHPDA-DYTAADFRRDATRAVESVLARGRLPIIAGGSNSY

ZmIPT7 (59) VTNKVTAAERQGVPHHLIDGVAPDA-DYTTADFCRDAVRAVESILERGRVPIIAGGSNRY

TaIPT5 (83) ATNKVSPSECAGVPHHLLGLVHPDE-DFSAADFRREAGSAARAAAARGYVPVVAGGSNSY

TaIPT6 (43) ATNKVSPSERAGVPHHLLGLVHPDE-DFTAADFRREAGSAARAAAARGYVPVVAGGSNSY

ZmIPT5 (93) ATNKVGLAERGRVPHHLLGVVHPDA-EFTAADFRREASRAADRAAARGRVPVIAGGSNSY

ZmIPT6 (91) ATNKVGPAERAAVPHHLLGVVHPDA-EFTAADFRREAAGAAARVASRGRVPIIAGGSNSY

OsIPT4 (95) VTNKVTEEECAGVPHHLISVARPDD-EFTAADFRREAARAAAGAVERGRLPIIAGGSNSY

ZmIPT4 (108) TTNKVTEQERAGVPHHLLGVARPDE-EFTAADFRREATRAARAITARGRLPIVAGGSNSY

OsIPT5 (95) ATNKATADERAGVPHHLIGVAHPDE-EFTAADFRRAASRAAAAVAARGALPIIAGGSNSY

TaIPT3 (45) TTNKVMLADRRGVAHHLLGAVRADAGELPPSSFRSLAAAKAADIAARGHVPVVAGGSNSL

OsIPT8 (73) TTNKVSLADRRGVPHHLLGAIRAEAGELPPSSFRSLAAAAAAGIASRGRVPVVAGGSNSL

OsIPT7 (76) TTNKVSLADRRGVPHHLLGAIRPEAGELPPSSFRSLAAATAASIAARRLVPVIAGGSNSL

ZmIPT8 (119) TTNKVAPADRRGVPHHLLGAIRPEAGELPPSTFRSLAAATAASIAARGRLPVVAGGSNSL

TaIPT8 (79) TTNKVTLADRRGVPHHLLGAVRPDAGELPPSSFRALAAAKAASIAARGLLPVVAGGSNSL

OsIPT6 (60) TTNKVSLQDRCGISHHLIASIPRNAGDFPVSFFRSAAKTTINCIARRGHTPIVVGGSNSL

TaIPT2 (111) IEALVEGDG-A-------------------AFRAAHDCLFLWLDAAPGMMEWYTGLRVDD

ZmIPT2 (112) IEALVEGDG-A-------------------AFRAAHDLLFVWVDAEQELLEWYAALRVDE

OsIPT2 (117) VEALVEGGG-G-------------------AFRAAHDCLFLWTDVAPGLLRWYTAARVDD

TaIPT7 (109) LEALLDGEP---------------------GFRRRYECCFLWVDSDTAVLERYVGDRVDC

ZmIPT7 (118) LEALLDGEPPA-------------------GFRGRYECCFLWVDSDLAVLDRYIGSRVDC

TaIPT5 (142) VEELVEGDR-R-------------------AFRERYDCCFLWVDVQLPVLRGFVARRVDD

TaIPT6 (102) VEELVEGDR-R-------------------AFRERYDCCFLWVDVQLPVLRGFVARRVDD

ZmIPT5 (152) VEELVEGDR-R-------------------AFRDRYECCFLWVDAQLPVLHGFVARRVDD

ZmIPT6 (150) VEELVEGDR-R-------------------AFRERYDCCFLWVDARLPVLHGFVARRVDE

OsIPT4 (154) VEELVEGDG-R-------------------AFRERYECCFLWVDVDLEVLRGFVARRVDE

ZmIPT4 (167) VEELVDGDR-A-------------------AFRDRYDCCFLWVDVQRAVLHGCVARRVDE

OsIPT5 (154) IEELVDGDR-R-------------------AFRDRYDCCFLWVDVQLPVLHGFVGRRVDD

TaIPT3 (105) IHAFLADRFDAH---APRDPFAAAATG--YRPALRFPCCLLWVDVDEAVLDEYLDRRVDD

OsIPT8 (133) IHALLADPIDA----APRDPFADADVG--YRPALRFPCCLLWVDVDDDVLDEYLDRRVDD

OsIPT7 (136) IHALLADHFDA----SAGDPFSPAAAFRHYRPALRFPCCLLWVHVDEALLDEYLDRRVDD

ZmIPT8 (179) IHALLADRLDAGAADPFSAPPQPAPPRWGRRPALRSPCCLLWVHVDAALLAEYLDRRVDD

TaIPT8 (139) IHALLAD---L--PDDAADPEDPFSLDRSYRPALRYPCCLLWVDVEEALLAEYLDRRVDD

OsIPT6 (120) IHGLLVDNFDS----SIVDPFGQLEVS--YRPTPRSQCCFLWVHVNEVILNEYLKHRVDD

TaIPT2 (151) MVRRGLVDEARAAFEEG-----ADYTRGVRRAIGLPEMHEYLRAEREG----AVGEAEMA

ZmIPT2 (152) MVARGLVSEARAAFGGAG----VDYNHGVRRAIGLPEMHAYLVAEREG----VAGEAELA

OsIPT2 (157) MVRRGLVGEARAGFVDGAG-AADYYTRGVRRAIGIPEMHGYLLAERSGG--EAADDGELA

TaIPT7 (148) MVEQGLVGEVRGFYR-TD----ADYSRGIRRAIGVPEMDTYFRMEAAGALDGDDG--RRA

ZmIPT7 (159) MLEQGLVREVRAFFRHDD----ADYSRGIRRAIGVPEMDMYFRMEAAGALDGDDDDQLRV

TaIPT5 (182) MCRRGLVDEVAAAFDPRR----TDYSRGVWRAIGVPELDAYLRSTG-----AGEEE--RA

TaIPT6 (142) MCRRGLVDEVAAAFDPRR----TDYSRGVWRAIGVPELDAYLRSTG-----AGEDE--RA

ZmIPT5 (192) MCRRGLVDEVAAAFDPRR----TDYSRGIWRAIGVPELDAYLRARGRG-HGHHHDQ----

ZmIPT6 (190) MCRRGLVDEVAAAFDPRR----TDYSRGIWRAIGVPEMDAYLRAGGHG-DGDGDEQEQRA

OsIPT4 (194) MCRRGLVREVAAAFDPRR----TDYSRGIWRAIGVPELDAYLRSRG-----DGADEEERA

ZmIPT4 (207) MRARGLVDEVAAAFDPRR----NDYSRGLWRAIGAPELDAYLRWPGPGVDGDAESEGERD

OsIPT5 (194) MCGRGMVAEIEAAFDPDR----TDYSRGVWRAIGVPELDAYLRS-----CAAAGGEEERA

TaIPT3 (160) MLGEGMVEELREYFATTSASERASHAG-LGKAIGVPEIGDYLAGRK--------------

OsIPT8 (187) MVGEGMVEELEEYFATTSASERASHAG-LGKAIGVPELGDYFAGRK--------------

OsIPT7 (192) MVDAGMVEELREYFATTTAAERAAHSG-LGKAIGVPELGDYFAGRK--------------

ZmIPT8 (239) MVRGGMVEELREYFAATTAAERAAHAAGLGRAIGVPELGACFAGRA--------------

TaIPT8 (194) MVGAGMVEELREYFAATTAQERAAHAAGLGKAIGVPELGEYLAGRR--------------

OsIPT6 (174) MVDAGLVEEIEEYFDTLSVNGHVPYVG-LGKTIGVPELSEYFTGRV--------------

TaIPT2 (202) AMLERAVREIKANTFRLVLQQAVKIRRLSTLEG-WDVRRVDATAVFAAMAEGLGG---HK

ZmIPT2 (204) AMLERAVREIKDNTFRLARTQAEKIRRLSTLDG-WDVRRIDVTPVFARKADGTEC---HE

OsIPT2 (214) AMLDGAVREIKANTYRLAATQVAKIRRLSALDG-WDVRRVDATVVVARMAEGAP----HR

TaIPT7 (201) GLLEAAVDEIRANTCRLVCSQLRKIHRLRCLPG---------------------------

ZmIPT7 (215) RLLAAAVNEIKANTCGLARRQLQKIHRLHGLQGWSDIHRLDVTEVLQLKVGNAGNPKAQR

TaIPT5 (231) SMLAAAVDEIKANTSRLACRQRGKIQRLSRMWR---VRRVDATEVFLKSGA------AAD

TaIPT6 (191) SMLAAAVDEIKANTSRLACRQRGKIQRLARMWR---VRRVDATEVFLKSGA------AAD

ZmIPT5 (243) -MLAAALHEIKANTSRLAVRQRGKIQRLERMWR---VRRVDATEVFLKRGL------AAD

ZmIPT6 (245) RMLAAALDEIKVNTSRLALRQRGKIQRLARMWR---VRRVDATEVFLKRGH------AAD

OsIPT4 (245) RMLAAAVAEIKLNTFRLACRQHRKIERLDRMWR---ARRVDATEVFRRRGH------AAD

ZmIPT4 (263) RLLAAAIEDIKSNTRRLSCRQRAKIQRLAKMWG---VRRVDATEVFRRRGD------EAD

OsIPT5 (245) RLLANAIEDIKANTRWLSCRQRAKIVRLDRLWR---IRRVDATEAFRRRGG------AAN

TaIPT3 (205) -SLTAAVDEIKANTRVLAAAQVGKIRRM--------------------------------

OsIPT8 (232) -SLDAAIDEIKANTRVLAARQVGKIRRMADVWG-WPIRRLDATATIRARLSGAGR-AAEA

OsIPT7 (237) -TFSEAIDDIKANTRVLAAAQVSKIRRMSDAWG-WPIHRLDASDTVRARLTRAGS-AAES

ZmIPT8 (285) -SFRAAIDDIKANTRDLAAAQVRKIRRMADAWG-WPIQRLDASATVRARLRGAGP-DAES

TaIPT8 (240) -SFRAAVDDIKANTRLLAAAQVSKIRRMADAWG-WPVRRLDASATIRARLAGAGP-AAES

OsIPT6 (219) -SCSDALSMMKTNTQILARSQVTKIHRMVDVWG-WHVHALDCTETILAHLTGSNK-YMED

TaIPT2 (258) EVWESTVWKPCQEMVRLFL-----------------------------------------

ZmIPT2 (260) LTWKKQVWEPCEEMVRAFLEPSLTAVPGVAVTEEGNAGVVATAAPAGDVVVPTGDVVTAV

OsIPT2 (269) ETWEAVVWKPCEEMVGRFLEAS----AAVDDDDNAAAGSPAALAPMTAACRLRAQLVQLQ

TaIPT7 (234) ------------------------------------------------------------

ZmIPT7 (275) DAWETDVVSPAARIVGMFLAVEGARDKDKDRFLLTTPKEVAVPGICTATADWFGQQLDMT

TaIPT5 (282) EAWQRLVAAPCIDAVRSFLLED------QECSMVAAAGKASVFASAAGNASVFAGRPATA

TaIPT6 (242) EAWQRLVAAPCIDAVRSFLLED------QECSMVAAAGKASVFASAAGNASVFAGRPATA

ZmIPT5 (293) EAWQRLVAAPCIDAVRSFLLED------QQEYSSMGTAGAMLPAAVAAAAV---------

ZmIPT6 (296) EAWQRLVAAPCIDAVRSFLLEE------QEYSSSMVTASMFASTAAAAV-----------

OsIPT4 (296) DAWQRLVAAPCIDAVRSFLFED------QERSSIAAGKPPLFAAGKATSGNISVFASAAA

ZmIPT4 (314) EAWQRLVAAPCIDAVRSFLR--------TDDAAATVASDLAVDGVVPVFAPAPAAAVAG-

OsIPT5 (296) EAWERHVAAPSIDTVRSFLHGE------FTTAAETTAAPVPPPPFLPMFALAAAGAGV--

TaIPT3 (232) ------------------------------------------------------------

OsIPT8 (289) AAWERDVRGPGLAAMRQFVGR---ADFNAAAVDQLAARSRR-----------QCLRGGMV

OsIPT7 (294) ASWERDVRGPGLATIRSFLADQSPPPRSEGTNDYLYAMETEPEPPPPPTLPPRLLRLPRM

ZmIPT8 (342) ACWERDVRAPGLAAIRSFLLELDGGSVVDGAV-----VEEVEP---------------RV

TaIPT8 (297) ASWERDVRGPGLAAIRAFLA----------------------------------------

OsIPT6 (276) LVWKRDVSDPGLATIQDFL-----------------------------------------

Additional file 7. Comparison of deduced protein sequences of selected *TaIPT* gene fragments with representative IPT proteins in maize and rice

Identical and conserved amino acids are shaded. Sequence segments not covered by TaIPTs fragments are not included.
