# Supplementary material for: Co-ordinate regulation of cytokinin gene family members during flag leaf and reproductive development in wheat
Source: BMC Plant Biol. 2012 Jun 6;12:78. doi: 10.1186/1471-2229-12-78 (PMC3410795; doi:10.1186/1471-2229-12-78)
Supplement: Additional file 8 — Comparison of deduced protein sequences of selectedTaCKXgene fragments with representative CKX proteins in maize and rice. [file 1471-2229-12-78-S8.doc]

TaCKX1 (1) -------LAKRKALMAIVYVLLVALITAASHAPDGAHGQTWHGDLAALAAAGKLRSDPNA

ZmCKX1 (1) -------MAVVYYLLLAGLIACSHALAAGTPALGDDRGRPWPASLAALALDGKLRTDSNA

OsCKX1 (1) -------MAVVYYLLLAGLIACSHALAAGTPALGDDRGRPWPASLAALALDGKLRTDSNA

TaCKX2 (1) MNKAREMTLIAALFVLGCFLQTVQTARADADALAWTPASPFRDELRDLGVAALIRDDAEA

ZmCKX5 (1) MARATTSTVAALCFLLSCVSATPSTLAASSAIIHDIIRG------LADTTAARVRTDAEA

OsCKX2 (1) --MKQEQVRMAVLLMLNCFVKATAPPPWPPSASSAS----FLDDLGDLGIAPLIRADEAG

TaCKX7 (1) --------MARTRLAAFLIGMASSFSVVAGQLRPMPAEVAFPVDLFALGIASKIRTDCNS

TaCKX8 (1) --------MARTRLAAFLIGMASFFSVVAGQLRPMPCRVAFPVDLFALGIASKIRTDCNS

ZmCKX7 (1) --------MARTRFVAVAALLTSFLSVAVGQPRPLPAAV-LPSDLFGLGIASRIRTDRNS

ZmCKX8 (1) -------MARRTRFVAVAALLASFLSVAAGHPRPLPAAG-LPGDLFGLGIASRIRTDSNS

TaCKX9 (1) ---------MPIARFTTFLIFTSFLSTIGH--LGAPAFGALDDDLLALDIVSKIHTDRSL

ZmCKX9 (1) -------MTARAAYLASFLIVTSFLPTS-------SSHSHVPAVTDALDIVSKISTEHDA

TaCKX3 (1) ------------------------------MMLAYMDRAAAGAAAERDALELTVVAADAA

ZmCKX10 (1) ------------------------------MMLAYMDRATAAAEPEDAGREP-ATTAGGC

TaCKX6 (1) -----------------MEIAMVCTR-VNLLILILSLCSPYKFIQSPMDFGPLNLLPTT-

ZmCKX6 (1) -----------------MEVAMVVSARASLLILVISLCSPYKFIQSPMDLGPLNLLPTTS

ZmCKX11 (1) -------------------MELNKVLYMYAATVALLLCSSANFIQSPSDVLGSVALQDP-

ZmCKX4 (1) -----------MTRCLMFMPPLFLVSSLISTVGLPVEPPAELLQLGGDVSGGRLSVDASD

HvCKX2 (1) -----------MRQLLLQYLKLFLLLGLGAVTAEHVLKHDVLASLGTLPLDGHFSFHDLS

TaCKX1 (54) TLAASTDFGNITAALP--------AAVLFPSSPADVAALLRAAHTTVA---WPYTISFRG

ZmCKX1 (54) TAAASTDFGNITSALP--------AAVLYPSSTADLVALLSAANSTPG---WPYTIAFRG

OsCKX1 (54) TAAASTDFGNITSALP--------AAVLYPSSTADLVALLSAANSTPG---WPYTIAFRG

TaCKX2 (61) TAPASTDFGNVTVA--------LAAAVLYPSCPADIAALLRASCARP----SPFPVSARG

ZmCKX5 (55) TARASTDFGTNATADDAAR----PAAVFYPSCAADIAALLRASSASA----SPFPVSARG

OsCKX2 (55) TARASADFGNLSVAGVGAPRLAAAAAVLYPSRPADIAALLRASCARP----APFAVSARG

TaCKX7 (53) TASAASDFGRIMEAAP--------EAVLHPATPADIAALIRFSTSSP----VPFPVSPRG

TaCKX8 (53) TASASSDFGRIMEAAP--------EAVLHPATPADIAALIRFSASSP----VPFPVSPRG

ZmCKX7 (52) TAKAATDFGQMARAAP--------EAVFHPATPADIAALVRFSASSV----APFPVAPRG

ZmCKX8 (53) TAKAATDFGQMVRAAP--------EAVFHPATPADIAALVRFSATSA----APFPVAPRG

TaCKX9 (50) TVRASSDFGHIVEATP--------NGVFHPVSPADIAALIRFSLYQQ----TPFTVAPRG

ZmCKX9 (47) TIKASMDFGHIVRAIP--------SGVFHPTSPSDIAALIRLSLSQP----KPFAVAPRG

TaCKX3 (31) ECAAARDFGGLVSARP--------AAVVRPASADDVASAIRAAART-----THLTVAARG

ZmCKX10 (30) AAAAATDFGGLASAMP--------AAVVRPASADDVASAIRAAALT-----PHLTVAARG

TaCKX6 (42) -IAASSDFGRILFHSP--------SAVLKPQSPRDISLLLSFLSAS-SLG--KVTVAARG

ZmCKX6 (44) TAAASSDFGRILFRAP--------AAVLRPQSPRDISMLLSFLSGSPSLS--RVTVAARG

TaCKX11 (1) -----------------------------------------ALSSAPPR--PRATVAARG

ZmCKX11 (41) TPSAAHDFGAVVSDAP--------VAVMQPGSPADIARLLSALSSAGPDRRPKAAVAARG

ZmCKX4 (50) IAEASRDFGGLSRAEP--------MAVFQPRAAGDVAGLVRAAFGSA----RGFRVSARG

HvCKX2 (50) AAAMDFGNLSSFPPVA--------VLHPGSVADIATTVRHVFLMGEH----SALTVAARG

TaCKX1 (103) RGHSVMGQALAPGGVVVDMPSLGGPSSAA--------------RINVSADGQYVDAGGEQ

ZmCKX1 (103) RGHSLMGQAFAPGGVVVNMASLGDAAAPP--------------RINVSADGRYVDAGGEQ

OsCKX1 (103) RGHSLMGQAFAPGGVVVNMASLGDAAAPP--------------RINVSADGRYVDAGGEQ

TaCKX2 (109) RGHSIRGQAAAPDGVVVDMPSLGRLGGGSS---------STASRLSVSVDGRYIDAGGEQ

ZmCKX5 (107) RGHSTRGQATAPGGVVVDMASLAVAAGRDETATTNASSTSASARLAVSVDGRYIDAGGEQ

OsCKX2 (111) CGHSVHGQASAPDGVVVDMASLGRLQGGG------------ARRLAVSVEGRYVDAGGEQ

TaCKX7 (101) QGHSVRGQSLAPGGVVVDMRTLGRGHHRINVSAD------------------YVDAGGEQ

TaCKX8 (101) QGHSVRGQSLAPGGVVVDMRMLGRGYHRINVSAD------------------YVDAGGEQ

ZmCKX7 (100) QGHSWRGQALAPGGVVVDMGSLGRG-PRINVSVA----------------EPFVDVGGEQ

ZmCKX8 (101) QGHSWRGQALAPGGVVVDMGSLGRG-PRINVSAAT-------------GAEPFVDAGGEQ

TaCKX9 (98) KGHSSRGQALAPGGIVVDMPSLGRGDHGH--------------RVNVSIDGMYVDVGGEQ

ZmCKX9 (95) RGHSARGQALAVGGVVVDMRSLHDHDHDHRAGHGRY-------RMNNAVPGAWVDVGGEQ

TaCKX3 (78) NGHSVAGQAMSEGGLVLDMRAGAASRR---LQMKLVS------PGGG---AAFADVPGGA

ZmCKX10 (77) NGHSVAGQAMAEGGLVLDMRSLAAPSRRAQMQLVVQC------PDGGGGRRCFADVPGGA

TaCKX6 (90) AGHSIHGQAQALDGIVVEMRCLP-----AEIELHRG---------GEG-DVSYADVSGGA

ZmCKX6 (94) AGHSIHGQAQAPDGIVVETRSLP-----GEMEFHHVR------GGGEG-RASYADVGGGV

TaCKX11 (18) VGHSLQGQAQARDGIVVETRSLPRTV--VVVAAPRAG------GEAT--ACAYADVGAGA

ZmCKX11 (93) AGHSLHGQAQARGGIVVETRALPRLV--EVVRRGDGD------GDGDG-GAAYADVGAGA

ZmCKX4 (98) HGHSISGQAQAPGGVVVDMGHGGAVARALPVHSP-------------ALGGHYVDVWGGE

HvCKX2 (98) HGHSLYGQSQAAGGIVIRMES----LRSVKMQVH-------------PGASPYVDASGGE

TaCKX1 (149) MWIDVLRATLERG-VAPRSWTDYLHLTVGGTLSNAGISGQTYRHGPQISNVLELDVITGY

ZmCKX1 (149) VWIDVLRASLARG-VAPRSWTDYLYLTVGGTLSNAGISGQAFRHGPQISNVLEMDVITGH

OsCKX1 (149) VWIDVLRASLARG-VAPRSWTDYLYLTVGGTLSNAGISGQAFRHGPQISNVLEMDVITGH

TaCKX2 (160) LWVDVLRAALAHG-LTPRSWTDYLHLTVGGTLSNAGISGQAFRYGPQISNVQELDVITGV

ZmCKX5 (167) LWVDVLHAALAHG-LTPRSWTDYLRLTVGGTLSNAGISGQAFRHGPQISNVLELDVVTGT

OsCKX2 (159) LWVDVLRASMAHG-LTPVSWTDYLHLTVGGTLSNAGISGQAFRHGPQISNVLELDVITGV

TaCKX7 (143) LWVDVLRATLKHG-LAPRAWTDHLQLTVGGTLSNAGIGGQAFRHGPQIANVHELDVVTGT

TaCKX8 (143) LWVDVLRATLKHG-LAPRAWTDYLQLTVGGTLSNAGIGGQAFRHGPQIANVHELDVVTGT

ZmCKX7 (143) LWVDVLRATLRHG-LAPRVWTDYLRLTVGGTLSNAGIGGQAFRHGPQIANVHELDVVTGT

ZmCKX8 (147) LWVDVLRATLRHG-LAPRVWTDYLRLTVGGTLSNAGIGGQAFRHGPQIANVHELDVVTGT

TaCKX9 (144) LWFDVLHATLKHG-LTPRVWTDYLRITVGGTLSNAGIGGQVFRHGPQISNVHELDVVTGT

ZmCKX9 (148) LWIDVLHATLEHD-LAPRVWTDYLHITVGGTLSNGGIGGQAFRHGPQISNVHELDVVTGT

TaCKX3 (126) LWEEVLHWAVSNHGLAPASWTDYLRLTVGGTLSNGGVSGQSFRYGPQVSNVAELEVVTGE

ZmCKX10 (131) LWEEVLHWAVDNHGLAPASWTDYLRLTVGGTLSNGGVSGQSFRYGPQVSNVAELEVVTGD

TaCKX6 (135) MWIELLEQSLK-AGLAPRSWTDYLYITIGGTLSNAGISGQTFKHGPQISNVLQLEVVTGR

ZmCKX6 (142) LWIELLERSLK-LGLAPRSWTDYLYLTVGGTLSNAGISGQTFKHGPQISNVLQLEVVTGR

TaCKX11 (68) LWVEVLEECLK-AGLAPLSWTDYLYLTVGGTLSNAGISGQAFKHGPQISNVLQLMLAAGN

ZmCKX11 (144) LWVEVLEECLR-AGLAPRSWTDYLYLTVGGTLSNGGISGQAFKHGPQISNVLQLEVVTGT

ZmCKX4 (145) LWVDVLNWTLSHGGLAPRSWTDYLYLSVGGTLSNAGISGQAFHHGPQISNVYELDVVTGK

HvCKX2 (141) LWINVLNKTLKYG-LAPKSWTDYLHLTVGGTLSNAGVSGQTFRHGPQISNVNELEIVTGR

TaCKX1 (208) GEMVTCSKSLNADLFDAVLGGLGQFGVIVRARIALEPAP-TRARWARLVYTDFAAFSADQ

ZmCKX1 (208) GEMVTCSKQLNADLFDAVLGGLGQFGVITRARIAVEPAP-ARARWVRLVYTDFAAFSADQ

OsCKX1 (208) GEMVTCSKQLNADLFDAVLGGLGQFGVITRARIAVEPAP-ARARWVRLVYTDFAAFSADQ

TaCKX2 (219) GEMVTCSKERNSDLFDAVLGGLGQFGVITRARIPLVPAP-TRARWVRLLYTGAAALTGDQ

ZmCKX5 (226) GDMVTCSKEKDADLFDAVLGGLGQFGIITRARIPLAPAP-ARARWLRLLYTGAADLTADQ

OsCKX2 (218) GEMVTCSKEKAPDLFDAVLGGLGQFGVITRARIPLAPAP-ARARWVRFVYTTAAAMTADQ

TaCKX7 (202) GEMVTCSRDKRKDLFFAALGGLGQFGIITRARIALESAP-KQVRWVRLAYSDVVAFTRDQ

TaCKX8 (202) GEMVTCSRDKRKDLFFAALGGLGQFGIITRARIALELAP-KQVRWVRLAYSDVVAFTRDQ

ZmCKX7 (202) GEMVTCSMDVNSDLFMAALGGLGQFGVITRARIRLEPAP-RRVRWVPLAYTDVATFTKDQ

ZmCKX8 (206) GEMVTCSMDVNSDLFMAALGGLGQFGVITRARIRLEPAP-KRVRWVRLAYTDVATFTKDQ

TaCKX9 (203) GDMITCSPGNNSDLFYGALGGLGQFGVITRARVGLERAP-KRVKWVRLAYTDVLPFTADQ

ZmCKX9 (207) GEMVSCSPGKNSDLFYAALGGLGQFGVITRARIALEPAP-RRVLWVRLAYSDVRSFTSDQ

TaCKX3 (186) GECRVCSHSAHPDLFFAVLGGLGQFGVITRARIPLSPAPQT-VKWARVVYASFAEYAADA

ZmCKX10 (191) GERRVCSPSSHPDLFFAVLGGLGQFGVITRARIPLHRAPQA-VRWTRVVYASIADYTADA

TaCKX6 (194) GETVTCSPTKNAELFSAVLGGLGQFGIITRARILLQEAPQK-VKWVRAFYDDFGTFTKDQ

ZmCKX6 (201) GEIVECSPSKEADLFNAVLGGLGQFGIITRARILLQEAPEK-VTWVRAFYDDLGAFTRDQ

TaCKX11 (127) GEVVTCSRTKSPDLFFAVLGGLGQFGIITRARILLQLPP---VRWVRAFYESFETFTKDQ

ZmCKX11 (203) GEVVTCSPTQSPELFFAVLGGLGQFGIITRARIPLQLAP---VRWVRAFYDSFETFTKDQ

ZmCKX4 (205) GEVVTCSETENPDLFFGVLGGLGQFGIITRARIALERAPQRQVRWIRALYSNFTEFTADQ

HvCKX4 (1) -----------------LSAAWASSASSPGTRIALEPAP-KMVRWIRVLYSDFASFTEDQ

TaCKX10 (1) ------------------------------------------VRWIRVLYLDFVSFTEDQ

HvCKX2 (200) GDIVTCSPEQNSDLFRAALGGLGQFGIITRARIALEPAP-QMVRWIRVLYLDFMSFTEDQ

TaCKX1 (267) ERLAAPGPGG----AFGPMSYLEGAVYVN--HSLAAGLRNSGG-----------FFTDAD

ZmCKX1 (267) ERLTAPRPGGG-GASFGPMSYVEGSVFVN--QSLATDLANTG------------FFTDAD

OsCKX1 (267) ERLTAPRPGGG-GASFGPMSYVEGSVFVN--QSLATDLANTG------------FFTDAD

TaCKX2 (278) EQLIDVERAN---ALSGLMDYVEGTVLAD--KGLIGSWRS----PSPSSSS--FCSEPDA

ZmCKX5 (285) ERLIADDERRG-GALAGLMDYVEGSVVTDLQQGLIGSWRSQ---PPPSSSS---FYSATD

OsCKX2 (277) ERLIAVDRAGGAGAVGGLMDYVEGSVHLN--QGLVETWRTQPQPPSPSSSSSSSFFSDAD

TaCKX7 (261) ELLISKHAS------EAGFDYVEGQVQLN--RTLTEGPKSTP------------FFSEAD

TaCKX8 (261) ELLISKQAS------EAGFDYVEGQVQLN--RTLTEGPKSTP------------FFSEAD

ZmCKX7 (261) EFLISNRAS------QVGFDYVEGQVQLS--RSLVEGPKSTP------------FFSGAD

ZmCKX8 (265) EFLISNRTS------QVGFDYVEGQVQLN--RSLVEGPKSTP------------FFSGAD

TaCKX9 (262) ELLISR---------GAGFDYVEGQVQLN--RTLTEGRRSSS------------FFSASE

ZmCKX9 (266) ELLISKRPAGG---CGSGFDYVEGQVQLN--RTLTEGRRSSS------------FFSAPE

TaCKX3 (245) EWLVTRPAE-------SAFDYVEGFAFVR-----SDDPVNGW--------PSVPIPAGAR

ZmCKX10 (250) EWLVTRPPD-------AAFDYVEGFAFVN-----SDDPVNGW--------PSVPIPGGAR

TaCKX6 (253) ELLVSMP---------DMVDYVEGFIVLN-----EQSLHS----------SSIAFPANMD

ZmCKX6 (260) ELLVSIP---------DSVDYVEGFMVLN-----ERSLHS----------SSIAFPASVD

TaCKX11 (184) ELLISMP---------EQVDYVEGFMVLD-----EHSIRS----------SSVAFPASID

ZmCKX11 (260) ELLVSMP---------ELVDYVEGFVVLN-----EQSLRS----------SSVAFPAQVD

ZmCKX4 (265) ERLISLG--------SRRFDYVEGFVVAA-EGLINNWRSSFFS----------------P

HvCKX4 (43) EALIST---------AKTFDYIEGFVIIN-RTGILNNWRTSFK----------------P

TaCKX10 (19) EMLISA---------EKTFDYIEGFVSIN-RTGILNNWRSSFN----------------P

HvCKX2 (259) EMLISA---------EKTFDYIEGFVIIN-RTGILNNWRSSFN----------------P

TaCKX1 (310) VARIVAVAAARNATTVYVIEATLNYDDATA-------AS--VEQELSSVLATLRHEEGLA

ZmCKX1 (312) VARIVALAGERNATTVYSIEATLNYDNATA-------AAAAVDQELASVLGTLSYVEGFA

OsCKX1 (312) VARIVALAGERNATTVYSIEATLNYDNATA-------AAAAVDQELASVLGTLSYVEGFA

TaCKX2 (327) AARVAKLTEEAGGVLYCLEGALYYGGTAG--------GEPDVEKRLEVLLRELRYARGFA

ZmCKX5 (338) AARIAALAEEAGGVLYFLEGAVYYGGASDT-------TAADVDKRVDVMLRELRYARGFA

OsCKX2 (335) EARVAALAKEAGGVLYFLEGAIYFGGAAGP-------SAADVDKRMDVLRRELRHERGFV

TaCKX7 (301) INRLAGLASETGSGAIYFIEAAMYYDETT---------APSVNQKLKMVLAQLSFVPGFV

TaCKX8 (301) INRLAGLASETGSGAIYFIEAAMYYDETT---------APSVNQKLKMVLAQLSFVPGFV

ZmCKX7 (301) LARLAGLASRTGPTAIYYIEGAMYYTEDT---------AISVDKKMKALLDQLSFEPGFA

ZmCKX8 (305) LARLAGLASRTGPTAIYYIEGAMYYTEDT---------AISVDKKMKALLDQLSFEPGFP

TaCKX9 (299) LARLTELALGTGSAAVYYIEGAMYYDDSS---------AATVDQKLEALLEELSFVPGFA

ZmCKX9 (309) LDQLARLAVATGSAAIYYIEGAMYYDDDTT-------TASSVNQKLETLLEELSFVPGLA

TaCKX3 (285) FDPSLLLAGE-SGPLLYCLEVALYQHPHQQPDDVDERMR--------EMMRRLKYVRGLE

ZmCKX10 (290) FDPSLLPAG--AGPVLYCLEVALYQYAHRPDDVDDDDEEDQAAVTVSRMMAPLKHVRGLE

TaCKX6 (289) FSPDFGTKG--RPKIYYCIEFAVHDYQHKNTNVDQVVEAIS---------VQMSHIASHL

ZmCKX6 (296) FSPDFGTRS--SPRIYYCVEFAVHHHHGYQKQSQAAVEAIS---------RRMSHMASQL

TaCKX11 (220) FSPDFGSEG--RKKVYYCIEFAVHDF----------------------------------

ZmCKX11 (296) FSPDFASGAGSNKVHYYCIEFAVHEFQQQDS----AADHVS---------GQLSYLRPHA

ZmCKX4 (300) QNPVKLSSLKHHSGVLYCLEVTKNYDDATAG------SVEQFVQDVDALLGELNFIPGTV

HvCKX4 (77) QDPVQASHFQSDGKVLYCLEMTKNFDPD---------EADIMEQEVGVLLSRLRYIQSTL

TaCKX10 (53) QDPERASQFETDRKVLFCLEMTKNFNPE---------EADIMEQEVHALLSQLRYTPPSL

HvCKX2 (293) QDPERASRFETDRKVLFCLEMTKNFNPE---------EADIMEQEVHALLSQLRYTPASL

TaCKX1 (361) FVRDASYLEFLDRVHGEEVALDKIGLW-RVPHPWLNVLVPRSRIADFDSGVFKGILQDTD

ZmCKX1 (365) FQRDVAYAAFLDRVHGEEVALNKLGLW-RVPHPWLNMFVPRSRIADFDRGVFKGILQGTD

OsCKX1 (365) FQRDVAYAAFLDRVHGEEVALNKLGLW-RVPHPWLNMFVPRSRIADFDRGVFKGILQGTD

TaCKX2 (379) FVQDVSYAGFLDRVRDGELKLPPSGLW-DVPHPWLNLFLPASRVLDFAAGVFHGILRGTG

ZmCKX5 (391) YVQDVSYEQFLDRVSAGERRLRGEGLW-DVPHPWLNLFLPRSRILDFAAGVFHGVLLPTR

OsCKX2 (388) FAQDVAYAGFLDRVHDGELKLRAAGLW-DVPHPWLNLFLPRSGVLAFADGVFHGILSRTP

TaCKX7 (352) FTKDVTYFQFLDRVRVEEVVLRSAGVW-DVPHPWLNLFVPRSRILDFDACVLKDILGGDN

TaCKX8 (352) FTKDVTYFQFLDRVRVEEAELRWAGVW-DVPHPWLNLFVPRSRILDFDAGVLKGILGGDN

ZmCKX7 (352) FTKDVTFVQFLDRVREEERVLRSAGAW-EVPHPWLNLFVPRSRILDFDDGVFKALLKDSN

ZmCKX8 (356) FTKDVTFVQFLDRVREEERVLRSAGAW-EVPHPWLNLFVPRSRILDFDDGVFKALLKDAN

TaCKX9 (350) FVRDVSYVQFLDRVGQEEQKLRSAGAW-DVPHPWLNLFVPRSRIHDFAAGVFDGVLRDTR

ZmCKX9 (362) FVRDVSYVDFLDRVGRDEQKLRSAGVW-DVPHPWLNIFVPRSRILDFDAGVFKGILKGTR

TaCKX3 (336) YAADVRYVEFLSRVNRVEEEARRSGSW-AAPHPWLNLFVSARDIADFDRAVLKGMLA--D

ZmCKX10 (348) FAADVGYVDFLSRVNRVEEEARRNGSW-DAPHPWLNLFVSARDIADFDRAVIKGMLA--D

TaCKX6 (338) YSVEVSYFDFLNRVRMEEMSLRSSGLW-EVHHPWLNMFVPKAGIRDLRDLLMDNISP--D

ZmCKX6 (345) YSVEVSYLDFLNRVRMEEVSLRSAGMWEEVHHPWLNMFVPKPGVAGFRDLLMDNVSP--D

ZmCKX11 (343) YSVQVAYLDFLNRVRMEEESLRSRGLW-DVPHPWLNLFVPRHGVARFKDLLMDTITQGDF

ZmCKX4 (354) FTTDLPYVDFLDRVHKAELKLRAKGMW-EVPHPWLNLFVPASRIADFDRGVFRGVLGGGT

TaCKX4 (22) FHTHVTYLEFLDRVHSSELKLRAQGLW-EVPHPWLNLLIPRSTIHRFATEVFGNILKDSN

HvCKX4 (128) FHTDVTYLEFLDRVHSSELKLRAQGLW-EVPHPWLN-LIPRSSIHRFARE----------

TaCKX10 (104) FHTDVTYMEFLDRVHSSEIKLRAKGLW-EVPHPWLNLIIPRSTVHTFAKQVFGKILEDNN

HvCKX2 (344) FHTDVTYIEFLDRVHSSEMKLRAKGLW-EVPHPWLNLIIPRSTIHTFAEQVFGKILEDNN

TaCKX1 (420) IAGPLV-----VYPLNKSR-WDDGMSAVTPAEE-------VFYAVSLLFSSV-------A

ZmCKX1 (424) IVGPLI-----VYPLNKSM-WDDGMSAATPSED-------VFYAVSLLFSSV-------A

OsCKX1 (424) IVGPLI-----VYPLNKSM-WDDGMSAATPSED-------VFYAVSLLFSSV-------A

TaCKX2 (438) AMG-----PVLVYPMNRDR-WDGNTSAVFPEEE------EVFYTVGILRSAV-------S

ZmCKX5 (450) TAGGGGGGPVLVYPMNRGK-WDGATSAVLPYDDGDGDGDEVFYTVGILRSAV-------A

OsCKX2 (447) AMG-----PVLIYPMNRNK-WDSNMSAVITDDDGD----EVFYTVGILRSAA-------A

TaCKX7 (411) PVG-----LILMYPMNTAK-WTSQMTAVKPPTVEDV-----FYMVGLLRSAL-------S

TaCKX8 (411) PVG-----LILMYPMNTAK-WNSRMTAMTPATGEDV-----FYTVGLLRSAL-------S

ZmCKX7 (411) PAG-----IILMYPMNKDR-WDDRMTAMTPATNDDD----VFYAVSFLWSAL-------S

ZmCKX8 (415) PAG-----IILMYPMNKDR-WDDRMTAMTPATDDDDN---VFYAVSFLWSAL-------S

TaCKX9 (409) PVG-----LILMYPMNRDR-WDDRMTVVTP----DED---VFYAVGLLRSAV-------A

ZmCKX9 (421) PVG-----LILMYPMNKDR-WDDRMTTATP----DED---VFYAVGLLRSAV-------A

TaCKX3 (393) GVDG----PMLIYPMLKSK-WDPNTSVALPEGE-------VFYLVALLRFCPGGS-----

ZmCKX10 (405) GIDG----PMLVYPMLKSK-WDPNTSVALPEGE-------VFYLVALLRFCRSG------

TaCKX6 (395) NFEG----LILIYPLLRDK-WGTNTSVVLPDSG--ST-EQVMYVVGILRSANP-----DE

ZmCKX6 (403) SFQG----LILIYPLLRDK-WDTNTSVVIPDSGPTAD-DPVMYVVGILRSANPGPEEDGD

ZmCKX11 (402) EFEG----PVLVYPLLANR-WWDGNTSAVVPAAP--D-GVMYVFS-VLRSTDP------A

ZmCKX4 (413) AGAG---GPILIYPMNKHRRWDPRSSVVTPDED-------VFYLVAFLRS-A-------L

TaCKX4 (81) N------GPILLYPVNRSK-WDNRTSVVIPEEE-------IFYLVGFLSSAP-------S

TaCKX10 (163) N------GPILLYPVNKSR-----------------------------------------

TaCKX1 (460) NDLKRLEAQ----NQKILRFCDLAGIGYKEYLGHYTAHGDWVRHF-GGKWK----RFVEM

ZmCKX1 (464) PNDLARLQEQ---NRRILRFCDLAGIQYKTYLARHTDRSDWVRHFGAAKWN----RFVEM

OsCKX1 (464) PNDLARLQEQ---NRRILRFCDLAGIQYKTYLARHTDRSDWVRHFGAAKWN----RFVEM

TaCKX2 (479) EGDLGRLEEQ---NEEILRFCEEAGIPCVQYLPYYAGQAGWEKKHFGPA---KWARFVER

ZmCKX5 (502) DGDLRRMEEQ---NAEVARFCEAAGIPCTQYLPSYATQADWAARHFGPAGSGRWDTFLRR

OsCKX2 (490) AGDVGRLEEQ---NDEILGFCEVAGIAYKQYLPYYGSQAEWQKRHFGAN---LWPRFVQR

TaCKX7 (453) ADELEHLQRE---NQSVLAFCDKEGIECKHYLPHYTSQDGWRRHFGAKWS-----NIAQL

TaCKX8 (453) ADELERLQRE---NQSVLAFCDKEGIECKQYLPYYTSQDGWRRHFGAKWS-----NIAQL

ZmCKX7 (454) ADDVPQLERW---NKAVLDFCDRSGIECKQYLPHYTSQDGWRRHFGAKWS-----RIAEL

ZmCKX8 (459) ADDVPQLERW---NKAVLDFCDRSGIECKQYLPHYTSQDGWRRHFGAKWS-----RIAEL

TaCKX9 (449) AGDLERLERE---NEAVLELCDRAGIGCKQYLPHHASQDGWRRHFGAKWG-----RVAAL

ZmCKX9 (461) AADLEQLERE---NAAVLEFCHREGIGYRQYLPSHASLDGWRRHFGEKWS-----RFAAL

TaCKX3 (436) ---GAAVEELVAQNGAIVDACRSSGYDFKTYFPHYRTEADWARHFG-AKWAR----FVDR

ZmCKX10 (447) ---GPAVDELVAQNGAILRACRANGYDYKAYFPSYRGEADWARHFGAARWRR----FVDR

TaCKX6 (442) GCSHHCLQELLRRHRHIADTAGVR-IGAKQYLAHHPTPAGWHQHFG-PRWER----FVER

ZmCKX6 (457) GCSHRCLHELLRSHRRIADAAEAR-LGAKQYLPHHPTPARWQQHLG-RRWER----FADR

ZmCKX11 (447) RCGRACVEGILEQHRRLADEACRR-LGAKQYLARQPSPAHWRDHFG-ASWDR----FVAR

ZmCKX4 (455) PGAPESLEALARQNRRVLDFCAEAGIGAKQYLPNHKAPGEWAEHFGAAR----WERFARL

TaCKX4 (120) ASGHGSVDHAVSLNDKILDFCDKAGVGMKQYLAPYTTQQQWKAHFGAR-----WETFERR

HvCKX2 (439) AIGPHSIEHTLNLNNQIIEFSNKASIGVKQYLPNYTTEPEWKAHYGAR-----WDAFQQR

TaCKX1 (511) KDKYDPKRLLSPGQDIFN-------------------

ZmCKX1 (517) KNKYDPKRLLSPGQDIFN-------------------

OsCKX1 (517) KNKYDPKRLLSPGQDIFN-------------------

TaCKX2 (533) KRKYDPKAILSRGQRIFTSPLA---------------

ZmCKX5 (559) KRKYDPMAILSRGQRIFSSPLLAS-------------

OsCKX2 (544) KSKYDPKAILSRGQGIFTSPLA---------------

TaCKX7 (505) KNKYDPHMIMSRGQRIFPLPTVPAASMAT--------

TaCKX8 (505) KAKYDPHAIMSRGQRIFPLPSVPAASTATT-------

ZmCKX7 (506) KARYDPRALLSPGQRIFPVPVEASGIASA--------

ZmCKX8 (511) KARYDPRALLSPGQRIFPVPVESSGIASA--------

TaCKX9 (501) KSTYDPRAILSPGQGIVPGRGGQHDARC---------

ZmCKX9 (513) KRKYDPRAILTPGQGIFFSAGHDLTDQQTGSDSRNVL

TaCKX3 (488) KARYDPLAILAPGQKIFARTP----------------

ZmCKX10 (500) KARYDPLAILAPGQKIFPRVPASVAV-----------

TaCKX6 (496) KNRFDPLSILGPGQGIFPK------------------

ZmCKX6 (511) KARFDPLRILGPGQGIFPRTAQD--------------

ZmCKX11 (501) KARFDPSHVLGPGQGIFPWTDSATASSV---------

ZmCKX4 (511) KAQFDPRAILAAGQGIFRPPGSPPLVADS--------

TaCKX4 (175) KHMYDPLAILAPGQRIFAKASLPMS------------

HvCKX2 (494) KNTYDPLAILAPGQKIFQKKPASLPLSSLQYLL----

Additional file 8. Comparison of deduced protein sequences of selected *TaCKX* gene fragments with representative CKX proteins in maize and rice

Identical and conserved amino acids are shaded. Sequence segments not covered by TaCKXs fragments are not included.
