# Supplementary material for: Co-ordinate regulation of cytokinin gene family members during flag leaf and reproductive development in wheat
Source: BMC Plant Biol. 2012 Jun 6;12:78. doi: 10.1186/1471-2229-12-78 (PMC3410795; doi:10.1186/1471-2229-12-78)
Supplement: Additional file 9 — Comparison of deduced protein sequence of TaZOG and their orthologues in selected protein sequences in maize andPhaseolus lunatus. [file 1471-2229-12-78-S9.doc]

TacZOG1 (1) ---------------------------NQLMHLSLLAASRGLSVHYAAPAAHLRQARSRL

TacZOG2 (1) -----MAVDSMESVALVAVPFPAQGHLNQLMHLSLLAASRGLSVHYAAPAAHLRQARSRL

ZmcZOG1 (1) -----MAVDTMESVAVVAVPFPAQGHLNQLLHLSLLLASRGLSVHYAAPPPHVRQARARV

ZmcZOG2 (1) -----MAVDTMESVAVVAVPFPAQGHLNQLLHLSLLLASRGLSVHYAAPPPHVRQARARV

ZOG1 (1) MALNDKSIPHETKVVVLLIPFPAQGHLNQFLHLSRLIVAQNIPVHYVGTVTHIRQATLRY

TaZOG1 (1) --------MAASELHFLLVPLVAQGHIIPMVDLARLIAARGPRVTVLTTPVNAARNRPAV

TaZOG3 (1) --------MAASELHFLLVPLVAQGHIIPMVDLARLLAARGPRVTVVTTPVNAARNRATV

ZmZOG1 (1) --------MAEAAPHFVLVPMLAQGHLLPMLDLARVLASHGARATVVLTPVNAARNRAFL

ZmZOG3 (1) ----------MAGLHFVLVPLLAQGHIIPMVDLARLLAGRGARVSVVTTPVNAARNGPVV

TaZOG2 (1) --------------HAVLVPFPAQGHVTPMMKLAKVLHCKGFHVTFVNTEYNHRRLVRSR

ZmZOG2 (1) --MGSIVADADRRPHAVCVPFPAQGHVTPMLKLAKVLHSRGFHITFVNSEFNHRRLLRSR

TacZOG1 (34) HGWDPQALGSVQFHDLGVSAYKSPAPDPAAPCPFPNHLLSMWQTFTTAARAPLAALLESL

TacZOG2 (56) HGWDPQALGSVRFHDLGVSAFESPAPDPAAPCPFPNHLMSMWQTFTTAARAPLAALLESL

ZmcZOG1 (56) HGWDPRALGSIRFHDLDVPPYDSPAPDLAAPSPFPNHLMPMFEAFAAAARAPLAALLQRL

ZmcZOG2 (56) HGWDPRALGSIHFHDLDVPAYDSPAPDLAAPSPFPNHLMPMFEAFAAAARAPLAALLQRL

ZOG1 (61) N----NPTSNIHFHAFQVPPFVS--PPPNPEDDFPSHLIPSFEAS-AHLREPVGKLLQSL

TaZOG1 (53) EGAARAGLR-VDLAELPFPGPRFGLPEGLENADQMVDQTIYVKFFQAIWGMAEPLEEYVR

TaZOG3 (53) ESARRAGLA-IELAEAPFPGPQVGLPEGLENLDQLLDQTTYLAFFQAIWKMAEPLQEYVR

ZmZOG1 (53) EQAAGAGLT-INFAELAFPGPALGLAAGCERVDMLQDLSLIVPFYDAVWLLAEPLEAYLL

ZmZOG3 (51) ESARRAGLD-VELAEVAFPGPGLGLPEGMENVDMVVEKEHFMPFFQATWKMDGPLEEYLR

TaZOG2 (47) GPDAVASLPGFRFATIPDGIP----TSDADSTQDPPSLCYYTMTTCLPHLKNLLRDLNGV

ZmZOG2 (59) GASALDGLPDFRFAAIPEGLP----PSDADATQDVPSLCRATMENCLPHFRSLLAELNSN

TacZOG1 (94) SGTHRRVVVVHDRLNCFAAVEAARLSNG----------EAFALQCVAISYNVGWMDTDHP

TacZOG2 (116) SATHRRVVVVHDRLNSFAAVEAARLSNG----------EAFALQCVAISYNIGWLDAEHP

ZmcZOG1 (116) STSYRRVAVVFDRLNPFAATEAARLANA----------DAFGLQCVAISYNVGWLDPGHR

ZmcZOG2 (116) STSYRRVAVVFDRLNPFAATEAARLANA----------DAFGLQCVAISYTVGWLDPGHR

ZOG1 (114) SSQAKRVVVINDSLMASVAQDAANISNVEN--------YTFHSFSAFNTSGDFWEEMGKP

TaZOG1 (112) ALPRRPDCLIADSCNPWTAGVCASLGIPRLVMHCP--SAYFLLAVHNLSTHGVYDRVGGD

TaZOG3 (112) ALPRRPDCLVADSCNPWTAGICTALGIPRLVLHCP--SAYFLLAVHNLSTHGVYDRVGDD

ZmZOG1 (112) SLPRMPDCLVSDSFMAWTASVARRHGILRFVVHFS--PASYVLAAHILETRGVYDRAA-D

ZmZOG3 (110) SLPRRPDCVIADSCNPWAARVCARHGIPRLVLHCP--SAYFLLATHCLSTHGVYGRVA-H

TaZOG2 (103) VGAPPVSCVVGDGVMSFCVDAAAELGVPCALFWTASACGFMGYRNFRFLLEEGLTPLEDE

ZmZOG2 (115) PDVPPVTCVVGDDVMSFTLEAAREVGVPCALFWTASACGYLGYRYYRDLMEKGIFPLKDA

TacZOG1 (144) LLLDNGLEFLPLDACMSKGVLEYVFQTEK--------------ESGGGRGAVPTAGMVMN

TacZOG2 (166) LLRDNGLRFHPIDACMSKEFLEYVFQTEK--------------EMQE-RGGVPTAGMVMN

ZmcZOG1 (166) LLSDYGLQFLPPDACMSREFVDLVFRMEE--------------EEQG----APVAGLVMN

ZmcZOG2 (166) LLSDYGLQFLPPDDCMSREFVDLVFRMEE--------------EEQG----APVAGLVMN

ZOG1 (166) PVGDFHFPEFPSLEGCIAAQFKGFRTAQY-------------------EFRKFNNGDIYN

TaZOG1 (170) DMEPFEVPDFPVPAVGNTATFRGFFQWPG-V---------EKEQQDVLDAEATADGLLVN

TaZOG3 (170) ELEPFEVPDFPVRAVGNTATFRGFFQHPG-A---------EKEQRDVLDAEVTADGLLIN

ZmZOG1 (169) DFEPFEVPEFPVRAVVSRATAQGVFQWPAGM---------ERFRRDTLDAEATADGILFN

ZmZOG3 (167) EMEPFEVPGFPVRAAGNVATFRGFFQWPG-M---------ESYERDVAEAEATADGLLIN

TaZOG2 (163) EQVTNGYLDTPVTQAHGMSEHMRLRDFSSFVRTTDPSDVLFNFLLHEVEQSDRATAIILN

ZmZOG2 (175) EQLTNGFLDTPTDWALGMSKHTRLKDFPSFVRSTDPDEFMFHFALKVTEQIVGADAVILN

TacZOG1 (190) TCRALEGDFMDAIAAHPVFKDQK--LFAVGPLNPLLDATARTPAKT-------------R

TacZOG2 (211) TCRALEGDFMDAIAAHPAFKDQN--LFAVGPLNPLLDASARTPAKT-------------R

ZmcZOG1 (208) TCRALEGEFLDVVAAQPPFQGQR--FFAVGPLNPLLLDADAPTTPPGQA----------R

ZmcZOG2 (208) TCRALEGEFLDAVAAQPPFQGQR--FFAVGPLNPLLLDADARTAP--------------R

ZOG1 (207) TSRVIEGPYVELLELFNGGKK----VWALGPFNPLAVEKKDSIG--------------FR

TaZOG1 (220) TFRGIESVFVDAYAAALGRRT-----WAVGPTCAS-SLGDADAKAGRGNRADVD-----A

TaZOG3 (220) TFRGVEGIFVDAYAVALGKRT-----WAIGPTCTS-GLGDADAMAGRGNRADVD-----V

ZmZOG1 (220) TCAALEDAFVERFASEVGKKI-----WAVGPLFLLGSGSDAGGMAGRGNRAAVD-----A

ZmZOG3 (217) TFRGLEGVFVDGYAAALGRKTTTTTCWAVGPTCASSGGLDAGATAGRGNRADVD-----V

TaZOG2 (223) TIDELEQTALDAMRAILP-----LPVYTIGPLNFLTEQLVSEGDGSGTLAAIRSSLWRED

ZmZOG2 (235) TFDELEQEALDAMRAMIPSS---ASIHTIGPLAFLAEEIVPRG---GPTDALGSNLWKED

TacZOG1 (235) HECMEWLDKQPPASVLYVSFGTTSSLLAEQIAELAAALKGSRQRFIWVLREADRADIFKE

TacZOG2 (256) HECMEWLDKQPPASVLYVSFGTTSSLLAEQIAELAAALKGSKQRFIWVLREADRADIFKE

ZmcZOG1 (256) HECLEWLDRQPPESVLYVSFGTTSCLHADQVAELAAALKGSKQRFVWVLRDADRADIYAE

ZmcZOG2 (252) HECLEWLDRQPPESVLYVSFGTTSCLHADQVAELAAALKGSKQRFVWVLRDADRADIYAE

ZOG1 (249) HPCMEWLDKQEPSSVIYISFGTTTALRDEQIQQIATGLEQSKQKFIWVLREADKGDIFAG

TaZOG1 (269) GHVVSWLDARPPASVLYISFGSIAKLPAKQVAELARGLEASGRPFVWAIKEAKADAAVQ-

TaZOG3 (269) GHVVSWLDARPPASVLYVSFGSIAQLPAKQLAELARGLEASGRPFVWAIKEAKADVGVK-

ZmZOG1 (270) DQIVSWLDARPAASVLYISFGSIGRLFPAQAAELAAGLEASRLPFIWSAKETAPG-----

ZmZOG3 (272) GLLLSWLDARPAASVLYVSFGSLAQLSLKQTVELARGLEASGRPFVWAIKEAKSSADVR-

TaZOG2 (278) QSCLEWLQGREPQSVVYVNYGSVTTMSKEELVEFAWGLANCGYDFLWIVRNDLVKGDAA-

ZmZOG2 (289) VSCFEWLHGRAPRSVVYVNYGSITVMTNEELVEFAWGLANSGHDFLWIIRPDLVNGDAA-

TacZOG1 (295) PGESLHDKLLSEFTKETEGTGLVITGWAPQLEILAHGATAAFMSHCGWNSTMESLSHGKP

TacZOG2 (316) PGESLHDKLLSEFTKETEGTGLVITGWAPQLEILAHGATAAFMSHCGWNSTMESLSHGKP

ZmcZOG1 (316) SGESRHAMFLSEFTRETEGTGLVITGWAPQLEILAHGATAAFMSHCGWNSTIESLSHGKP

ZmcZOG2 (312) SGDSRHAKFLSEFTRETEGTGLVVTGWAPQLEILAHGATAAFMSHCGWNSIIESLSHGKP

ZOG1 (309) -SEAKRYELPKGFEERVEGMGLVVRDWAPQLEILSHSSTGGFMSHCGWNSCLESITMGVP

TaZOG1 (328) -AL----LDDEGFEERVKDRGLLVRGWAPQVTILSHPAVGGFLTHCGWNATLEAISHGVP

TaZOG3 (328) -AL----LDDEGFEERVKDRGLVVRGWAPQVTILSHPAVGGFLTHCGWNATLEAISHGMP

ZmZOG1 (325) --------LDAEFEERVKDRGLVVHGWAPQMTILSHPAVGGFLTHCGWNSILESLCYGVP

ZmZOG3 (331) -AW----LLAERFEERVRDRGLLVRGWAPQVTILSHPAVGGFLSHCGWNASLEAITHGVP

TaZOG2 (337) -VLP------PEFLEATKDR-CLLASWCPQEAVIRHEAVGAFLTHCGWNSTMEGLCAGVP

ZmZOG2 (348) -VLP------PEFLEAIRGR-GHLASWCPQEVVLRHEAVGVFLTHCGWNSTMESLCAGVP

TacZOG1 (355) ILAWPMHSDQPWDAELLCKYVKAGLLVRPWEKHS------------EVVPAAAIQEVIEE

TacZOG2 (376) ILAWPMHSDQPWDAELLCKYLKAGLLVRPWEKHS------------EVVPAAAIQEVIEE

ZmcZOG1 (376) VLAWPMHSDQPWDSELLCKYFKAGLLVRPWEKHA------------EIVPAQAIQKVIEE

ZmcZOG2 (372) VLAWPMHSDQPWDSELLCNYFKAGLLVRPWEKHA------------EIIPAQAIQKVIEE

ZOG1 (368) IATWPMHSDQPRNAVLVTEVLKVGLVVKDWAQRN------------SLVSASVVENGVRR

TaZOG1 (383) ALTWPNFADQFCSERLLVDVLGVGVRSGVKLPVMNVPAEAEG----VQITSGDVEKVVAE

TaZOG3 (383) VLTWPCFADQFCSERLLVDVLGVGVRSGVKVPAKNVPEEAEG----VQVPSGDMEKAIAE

ZmZOG1 (377) LMTWPLFVDQFLNEALVVDVLGAGVRSGAKVPVTHVTVVKPGEVLEVQVWRDGVERAVTD

ZmZOG3 (386) VLTWPNFADQFCSERLLVDVLGVGVRSGVKLPPMSLPDEAEG----VQVTSADVEKAVAE

TaZOG2 (389) MLCWPFFAEQQTNSRYACMEWGVGMEVGDDVRRE------------------VVDARIRE

ZmZOG2 (400) MLCWPFFAEQQTNCRYTCVEWGVAMEIGQDVRRE------------------AVEEKIRE

TacZOG1 (403) AMLT-DKGMAVRQRAKVLGEAVRAAVADGGSSSKGLDDFVAYVTR---------------

TacZOG2 (424) AMLT-DKGMAVRQRAKVLGEAVRAAVADGGSSSKGLDDFVAYVTR---------------

ZmcZOG1 (424) AMLS-DSGMAVRQRAKELGEAVRASVADGGNSRKDLDDFIGYITR---------------

ZmcZOG2 (420) AMLS-DSGMAVRQRAKELGEAVRASVADGGNSRKDLDDFIGYITR---------------

ZOG1 (416) LMET-KEGDEMRQRAVRLKNAIHRSMDEGGVSHMEMGSFIAHISK---------------

TaZOG1 (439) LMDDGPEGAARRSRAKELAAEARAAMEEGGSSYTDLEDMICYASELSRKRS---------

TaZOG3 (439) LMDGGSEGMVRRSRAKEVAAEMRVAMEEGGSSYSDLTDMIHYVSELSRKAA---------

ZmZOG1 (437) LMDEGPAGAARRARAKELGQQMRAAMAKGGSSDTDVRNLVRHVVEVARKKEEHEDTALAG

ZmZOG3 (442) LMAVGADGTARRARAKELAAKAKAAMEEGGSSYADLDDMLRHVAELNMKKSHEEGTGFST

TaZOG2 (431) VMGGEVGREMRRKAAEWKEVASRSTAQHGGRSLANLESLLKDVLK---------------

ZmZOG2 (442) AMGGEKGMEMQRRAGEWQQIGLRATRPRG-RSYANLDKLVADVLLSGTSGKSS-------

Additional file 9. Comparison of deduced protein sequence of TaZOG and their orthologues in selected protein sequences in maize and *Phaseolus lunatus*
