# Supplementary material for: Co-ordinate regulation of cytokinin gene family members during flag leaf and reproductive development in wheat
Source: BMC Plant Biol. 2012 Jun 6;12:78. doi: 10.1186/1471-2229-12-78 (PMC3410795; doi:10.1186/1471-2229-12-78)
Supplement: Additional file 10 — Comparison of deduced protein sequence ofTaGLUgenes and their orthologues in maize, rice, and barley. [file 1471-2229-12-78-S10.doc]

TaGLU1a (61) GPVFTKLKPWQIPKRDWFDKDFLFGASTSAYQIEGAWNEDGKGPSTWDHFCHTYPERISD

ZmGlu1 (61) QNGVQMLSPSEIPQRDWFPSDFTFGAATSAYQIEGAWNEDGKGESNWDHFCHNHPERILD

TaGlu4 (1) ----------------------------------------GRGPSIWDAFAH-VPGNIAG

Os-B7F7K7 (37) ADHQEAAGITGGLSRRSFPAGFVFGTAASAYQVEGMALKDGRGPSIWDAFVK-TPGEIAN

Hv-ACF07998 (32) -----EIGNTGGLSRQGFPAGFVFGTAASAYQVEGMARQGGRGPCIWDAFVA-IPGMIAG

TaGlu3 (1) -------ACAEAIRRADFPPGFTFGTASSAYQYEGAVNEGQRGPTIWDTLAS-RPGRVID

OsGlu25 (8) HILVSFSACVEAISRADFPPGFIFGTASSAYQYEGAVNEGQRGPTIWDTLTK-RPGRVID

TaGLU1a (121) RTNGDVAANSYHLYEEDVKALKDMGMKVYRFSIAWSRILPDGTGK--VNQAGIDYYNKLI

ZmGlu1 (121) GSNSDIGANSYHMYKTDVRLLKEMGMDAYRFSISWPRILPKGTKEGGINPDGIKYYRNLI

TaGlu4 (20) NQNADVTTDQYHRYKEDVNLMKGLGFDAYRFSISWSRIFPDGTGK--VNQEGVAYYNNLI

Os-B7F7K7 (96) NATADVTVDEYHRYKEDVNIMKSMGFDAYRFSISWSRIFPTGTGK--VNWKGVAYYNRLI

Hv-ACF07998 (86) NGTADVTVDEYHRYKEDVGIMKNMGFDAYRFSIIWSRIFPDGTGK--VNQEGVDYYNRLI

TaGlu3 (53) FSNADVAVDHYHRYKEDVDLMKDIGVDAYRFSISWSRIFPNGTGK--PNEEGLSYYNSLI

OsGlu25 (67) FSNADVAVDHYHRYKEDVELMNDIGMDAYRFSISWSRIFPNGTGE--PNEEGLSYYNSLI

TaGLU1a (179) NSLIDNDIVPYVTIWHWDTPQALEDKYGGFLNR---KIVDDYKQFAEVCFKNFGDRVKNW

ZmGlu1 (181) NLLLENGIEPYVTIFHWDVPQALEEKYGGFLDKSHKSIVEDYTYFAKVCFDNFGDKVKNW

TaGlu4 (78) NYLLQKGITPYINLYHYDLPLALEKKYGGWLNA---KIVGLFADYAEFCFKTFGDRVKHW

Os-B7F7K7 (154) NYMLKIGITPYANLYHYDLPEALEVQYGGLLNR---KIVEAFADYAEFCFKTFGDRVKNW

Hv-ACF07998 (144) DYMLQQGITPYANLYHYDLPLALHQQYLGWLSP---KIVGAFADYAEFCFKVFGDRVKNW

TaGlu3 (111) DVLLDKGIQPYVTLFHWDLPQALEDKYGGWLNS---QIVEDFVHYASTCFKEFGDRVKHW

OsGlu25 (125) DALLDKGIEPYVTLFHWDLPQALEDRYGGWLNS---EIIEDFVQYAFTCFKEFGDRVKHW

TaGLU1a (236) FTFNEPHTYCCFSYGEGIHAPGRCSPGMDCAVPKGDSLREPYTAGHHILLAHAEAVELFK

ZmGlu1 (241) LTFNEPQTFTSFSYGTGVFAPGRCSPGLDCAYPTGNSLVEPYTAGHNILLAHAEAVDLYN

TaGlu4 (135) FTFNEPRIVALLGYDVGSNAPGRCSKCAAGG----NSATEPYIVAHNFLLAHAYAVARYR

Os-B7F7K7 (211) MTFNEPRVVAALGYDDGNFAPGRCTKCTAG-----NSATEPYIVAHHLILSHASAVQRYR

Hv-ACF07998 (201) FTFNEPRVVAALGYDNGFHAPGRCSKCPAGG----DSRTEPYIVTHNIILSHAAAVQRYR

TaGlu3 (168) ITVNEPHNFAIDGYDFGIQAPGRCSIISHLFCQEGTSSTEPYIVAHNILLAHAGVFHAYK

OsGlu25 (182) ITFNEPYNFAIDGYDLGIQAPGRCSILSHVFCREGKSSTEPYIVAHNILLAHAGAFRAYE

TaGLU1a (296) ACYNKHGDSKIGMAFDVMGYEPFQDSFLDDQARERSIDYNLGWFLEPVVRGDYPFSMRSL

TaGlu2 (1) --------------------------------------------LEPVVRGDYPFSMRSL

ZmGlu1 (301) KHY-KRDDTRIGLAFDVMGRVPYGTSFLDKQAEERSWDINLGWFLEPVVRGDYPFSMRSL

TaGlu4 (191) TKHQAAQKGKVGIVLDFVWYEALTNSTEDQAAAQRARDFHVGWFVDPLINGHYPQSMQDL

Os-B7F7K7 (266) HKYQHIQKGKIGILLDFVWYEGLTNSTADQAAAQRSRDFHVGWFLHPIIYGEYPKSLQVI

Hv-ACF07998 (257) EKYQPHQKGRIGILLDFVWYEPHSDSNADQAAAQRARDFHIGWFLDPITNGRYPSSMLKI

TaGlu3 (228) QHFKKKQGGLIGIALDSKWYEPLSDVDEDREAAARAMDFELGWFLDPLMFGRYPASMQKL

OsGlu25 (242) QHFKNEQGGLIGIALNSRWYEPFSNADEDTEAAARAMDFELGWFLDPLMFGHYPPSMQKL

TaGLU1a (356) IGDRLPKFTKEEQEKLASSCDIMGLNYYTSRFSKHIDISSDFTPKLNTDDAYASSETKGS

TaGlu2 (17) ARERLPFFKDEQKEKLAGSYNMLGLNYYTSRFSKNIDISPNYSPVLNTDDAYASQEVNGP

ZmGlu1 (360) ARERLPFFKDEQKEKLAGSYNMLGLNYYTSRFSKNIDISPNYSPVLNTDDAYASQEVNGP

TaGlu4 (251) VKERLPRFTPSEAKLLKGSADYIGINQYTASLMKDQKL--LQQTPTSYSADWQVTYAFER

Os-B7F7K7 (326) VKERLPKFTADEVHMVKGSIDYVGINQYTAYYVRDQQP--NATTLPSYSSDWHAAPIYER

Hv-ACF07998 (317) VGNRLPGFSADESRMVKGSIDYVGINQYTSYYMKDPGA--WNQTPVSYQDDWHVGFVYER

TaGlu3 (288) VGDRLPQFSSHESQLVSGSLDFVGINHYTTLYARNDRMRVRKLVMNDASTDAAVISTAYR

OsGlu25 (302) AGDRLPQFSTHASKLVSGSLDFVGINHYTTLYARNDRLRIRKLVMDDASTDSAVIPTAYR

TaGLU1a (416) DGNDIGPITGTYWIYMYPKGLTDLLLIMKEKYGNPPIFITENGIADVDSDPT---MTDPL

TaGlu2 (77) DGKPIGPPMGNPWIYMYPEGLKDLLMIMKNKYGNPPIYITENGIGDVDTKETPLPMEAAL

ZmGlu1 (420) DGKPIGPPMGNPWIYMYPEGLKDLLMIMKNKYGNPPIYITENGIGDVDTKETPLPMEAAL

TaGlu4 (309) NGKPIGPKANSNWLYIVPSGMYKCVHYLKEKYGNPPIVITEN--GMDEPGN--LTREQYL

Os-B7F7K7 (384) DGVPIGPRANSDWLYIVPWGLYKAVTYVKEKYGNPTMFLSEN--GMDDPGN--VTIAQGV

Hv-ACF07998 (375) NGVPIGPRANSDWLYIVPWGMNKAVTYVKERYGNPTMILSEN--GMDQPGN--VSIADGV

TaGlu3 (348) HGKEIGETAASNW-----------------------------------------------

OsGlu25 (362) HGKKIGETAASSWLHIVPWGMFKLMKHVKEKYGNPPVVITEN--GMDDANHPFSRLEDVL

TaGLU1a (473) DDWKRLDYLQRHISAVKDAIDQ-GADVRGHFTWGLIDNFEWSLGYSSRFGLVYIDKKDGN

TaGlu2 (137) NDYKRLDYIQRHIATLKESIDL-GSNVQGYFAWSLLDNFEWFAGFTERYGIVYVDRNNNC

ZmGlu1 (480) NDYKRLDYIQRHIATLKESIDL-GSNVQGYFAWSLLDNFEWFAGFTERYGIVYVDRNNNC

TaGlu4 (365) RDATRVRFYRSYLTELKKAIDN-GANVLGYFAWSLLDNFEWSLGYSSKFGIVYVDFSSTL

Os-B7F7K7 (440) HDTTRVAYYRSYITKLKEAIDD-GANCIGYFAWSLLDNFEWKLGYTSRFGLVYVDFR-TL

Hv-ACF07998 (431) HDTVRIRYYRDYITELKKAIDN-GARVAGYFAWSLLDNFEWRLGYTARFGIVYVDFN-TL

OsGlu25 (420) QDDKRIQYHNDYMSNLLDAIRKEGCNVHGYFVWSLLDNWEWNSGYTVRFGLYYIDYKNNL

TaGLU1a (532) KRKLKKSAKWFAKFNSVPKRLLKTTNNNATVTVTSVSV----------------------

TaGlu2 (196) TRYMKDSAQVLERVNTAKKPNNEDLTQLKNRGP-VGEPIKRWFEPKFLFFPQKSWRI-HT

ZmGlu1 (539) TRYMKESAKWLKEFNTAKKPSKKILTPA--------------------------------

TaGlu4 (424) DRHPKASAYWFRDLL---------------------------------------------

Os-B7F7K7 (498) RRYPKMSAYWFRDLVSSKN-----------------------------------------

Hv-ACF07998 (489) KRYPKDSALWFKNMLSEKKRS---------------------------------------

OsGlu25 (480) TRIPKASVQWFSQVLAQKTAII--------------------------------------

Additional file 10. Comparison of deduced protein sequence of *TaGlu* genes and their orthologues in maize, rice, and barley.
